# SNORA58 Facilitates Radioresistance via Suppressing JNK1‐Mediated Ferroptosis in Esophageal Squamous Cell Carcinoma

**DOI:** 10.1002/advs.202508515

**Published:** 2025-07-26

**Authors:** Yinli Zheng, Fangyi Liu, Yuhua Huang, Yanfen Feng, Xia Yang, Jinjun Wu, Xin Yang, Xuanhao Lin, Lives Jiang, Tingting Zeng, Yan Li, Xinyuan Guan, Yuanyuan Wang, Chunyan Chen, Jingping Yun

**Affiliations:** ^1^ State Key Laboratory of Oncology in South China Guangdong Provincial Clinical Research Center for Cancer Sun Yat‐sen University Cancer Center Guangzhou 510060 P. R. China; ^2^ Department of Pathology Sun Yat‐sen University Cancer Center Guangzhou 510060 P. R. China; ^3^ Joint Laboratory for Translational Cancer Research of Chinese Medicine of the Ministry of Education of the People's Republic of China International Institute for Translational Chinese Medicine Guangzhou University of Chinese Medicine Guangzhou 510006 P. R. China; ^4^ Department of Pathology National Cancer Center National Clinical Research Center for Cancer Cancer Hospital & Shenzhen Hospital Chinese Academy of Medical Sciences and Peking Union Medical College Shenzhen 518000 P. R. China; ^5^ Department of Pathology Shantou Central Hospital Shantou Guangdong 515041 P. R. China; ^6^ Department of Pathology Affiliated Cancer Hospital and institute of Guangzhou Medical University Guangdong 510095 P. R. China; ^7^ Department of Clinical Oncology State Key Laboratory for Liver Research The University of Hong Kong Hong Kong 852 P. R. China; ^8^ Department of Clinical Oncology The University of Hong Kong‐Shenzhen Hospital Shenzhen 518053 P. R. China; ^9^ Department of Radiology Oncology Sun Yat‐sen University Cancer Center Guangzhou 510060 P. R. China

**Keywords:** esophageal squamous cell carcinoma, ferroptosis, JNK1, radioresistance, SNORA58

## Abstract

Radioresistance represents a substantial challenge in cancer treatment, particularly in esophageal squamous cell carcinoma (ESCC), where the underlying molecular mechanisms remain incompletely understood. Small nucleolar RNAs (snoRNAs), primarily located in the nucleolus, are noncoding RNAs whose roles in ESCC radiotherapy are unclear. In this study, an upregulated snoRNA, SNORA58 is identified in ESCC via a snoRNA PCR array. Furthermore, based on multicenter data, SNORA58 is established as a promising biomarker for predicting response to neoadjuvant chemoradiotherapy (nCRT). Patients with high SNORA58 expression levels presented a lower likelihood of achieving a complete response to nCRT and poorer clinical outcomes. Functionally, SNORA58 enhances cancer cell resistance to radiotherapy without affecting chemotherapeutic sensitivity. Mechanistically, SNORA58 stabilizes CTCF by inhibiting its ubiquitin‐mediated degradation, leading to JNK1 downregulation and subsequent inactivation of the JNK signaling pathway; this disrupts intracellular iron homeostasis, thereby alleviating radiotherapy‐induced ferroptosis. Notably, the administration of a JNK signaling activator significantly restored the radiosensitivity of high‐SNORA58 ESCC cells both in vitro and in vivo. These findings elucidate the first demonstration of SNORA58 as a critical regulator of radioresistance in ESCC and reveal a novel link between snoRNAs and ferroptosis in this specific context, suggesting potential therapeutic strategies for managing ESCC.

## Graphical abstract

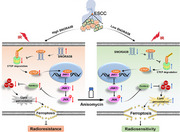

## 1 Introduction

The heterogeneity in radiosensitivity among patients with esophageal cancer (EC) is a critical factor influencing the therapeutic effectiveness of radiotherapy. Esophageal squamous cell carcinoma (ESCC) is the predominant histological subtype of EC in Eastern Asia, particularly in China, which has the highest incidence and mortality rates of ESCC worldwide.^[^ ^1^, ^2^ ^]^ To date, although the molecular mechanisms underlying radioresistance in ESCC have been extensively studied, mainly involving dysregulation of DNA damage repair pathways, the capacity to eliminate reactive oxygen species (ROS), epithelial‒mesenchymal transition, programmed cell death processes, the presence of cancer stem cells, remodeling of the tumor microenvironment, and aberrant expression of noncoding RNAs,^[^ ^3^, ^4^, ^5^, ^6^, ^7^, ^8^ ^]^ the translation of these findings into clinical applications remains limited. Therefore, identifying biomarkers with clinical utility for predicting radiotherapy response and guiding treatment decisions is of paramount importance.

Small nucleolar RNAs (snoRNAs) represent a diverse and highly conserved group of noncoding RNAs (ncRNAs) ranging in size from 60 to 300 nucleotides and are predominantly localized in the nucleoli of eukaryotic cells.^[^ ^9^, ^10^ ^]^ Based on their conserved structural features, snoRNAs are primarily categorized into two subtypes: C/D box snoRNAs and H/ACA box snoRNAs. These subtypes canonically function in guiding 2'‐O‐methylation and pseudouridylation modifications of ribosomal RNA (rRNA) or messenger RNA (mRNA), respectively. Owing to their stability and targetability, snoRNAs serve as promising biomarkers for diagnosis, therapeutic efficacy evaluation, and treatment across various human diseases, including cancers. Numerous snoRNAs have been identified as critical regulators of cancer malignancy. For example, SNORD17 promotes MDM2‐mediated p53 ubiquitination to facilitate metastasis in hepatocellular carcinoma^[^ ^11^ ^]^; SNORD42A directs 2'‐O‐methylation of 18S rRNA to sustain cell proliferation in acute myeloid leukemia^[^ ^12^ ^]^; the deletion of SNORD50A and SNORD50B enhances tumorigenesis in human cancers^[^ ^13^ ^]^; SNORA38B sensitizes immune checkpoint blockade by remodeling the tumor microenvironment in non‐small cell lung cancer^[^ ^14^ ^]^; snoRNA‐93 is processed into a microRNA‐like RNA to promote cell invasion in breast cancer^[^ ^15^ ^]^; SNORA18L5 increases the risk of HBV‐related hepatocellular carcinoma by reducing p53 levels through altering ribosomal protein localization^[^ ^16^ ^]^; and SNORA42 functions as an oncogene and prognostic biomarker in colorectal cancer.^[^ ^17^ ^]^ To date, only a limited number of studies have explored snoRNAs in ESCC. For example, SNORA42 has been shown to promote ESCC development by activating the DHX9/p65 axis,^[^ ^18^ ^]^ and SNORD12B activates the AKT‐mTOR‐4EBP1 signaling pathway to enhance malignant phenotypes in ESCC.^[^ ^19^ ^]^ Radiotherapy represents one of the primary treatment modalities for advanced ESCC. However, to our knowledge, no relevant studies have investigated the roles or applications of snoRNAs in ESCC radiotherapy.

Ferroptosis is a newly identified regulated cell death process that relies on iron‐mediated oxidative damage to induce cell membrane injury.^[^ ^20^, ^21^ ^]^ Ferroptosis has been recognized as an essential form of cell death induced by ionizing radiation (IR) in cancers, independent of the degree of apoptosis caused by DNA double‐strand breaks.^[^ ^22^, ^23^, ^24^ ^]^ Accumulating studies have elucidated the molecular mechanisms underlying IR‐induced ferroptosis, which involve the overproduction of reactive oxygen species (ROS) to trigger lipid peroxidation, disruption of intracellular iron homeostasis, upregulation of ASCL4 expression to promote the biosynthesis of polyunsaturated fatty acids (PUFAs), and depletion of GSH to abolish GPX4‐mediated defense.^[^ ^25^ ^]^ Nonetheless, the role of snoRNAs in regulating IR‐induced ferroptosis remains largely unexplored.

In the present study, we identified SNORA58 as a promising signature for predicting the prognosis and response to nCRT in ESCC patients. Functionally and mechanistically, we demonstrated that SNORA58 mitigates IR‐induced ferroptosis by disrupting intracellular iron homeostasis through the SNORA58/CTCF/JNK regulatory axis, consequently increasing radioresistance. Moreover, the combination of a JNK signaling activator with IR significantly resensitizes cancer cells to radiotherapy. These findings highlight the clinical importance of SNORA58 in ESCC radiotherapy and pave the way for precision treatment strategies.

## 2 Results

### 2.1 Aberrant SNORA58 Overexpression Indicates Unfavorable Outcomes and Poor Therapeutic Efficacy of nCRT in ESCC Patients

**Figure 1:**
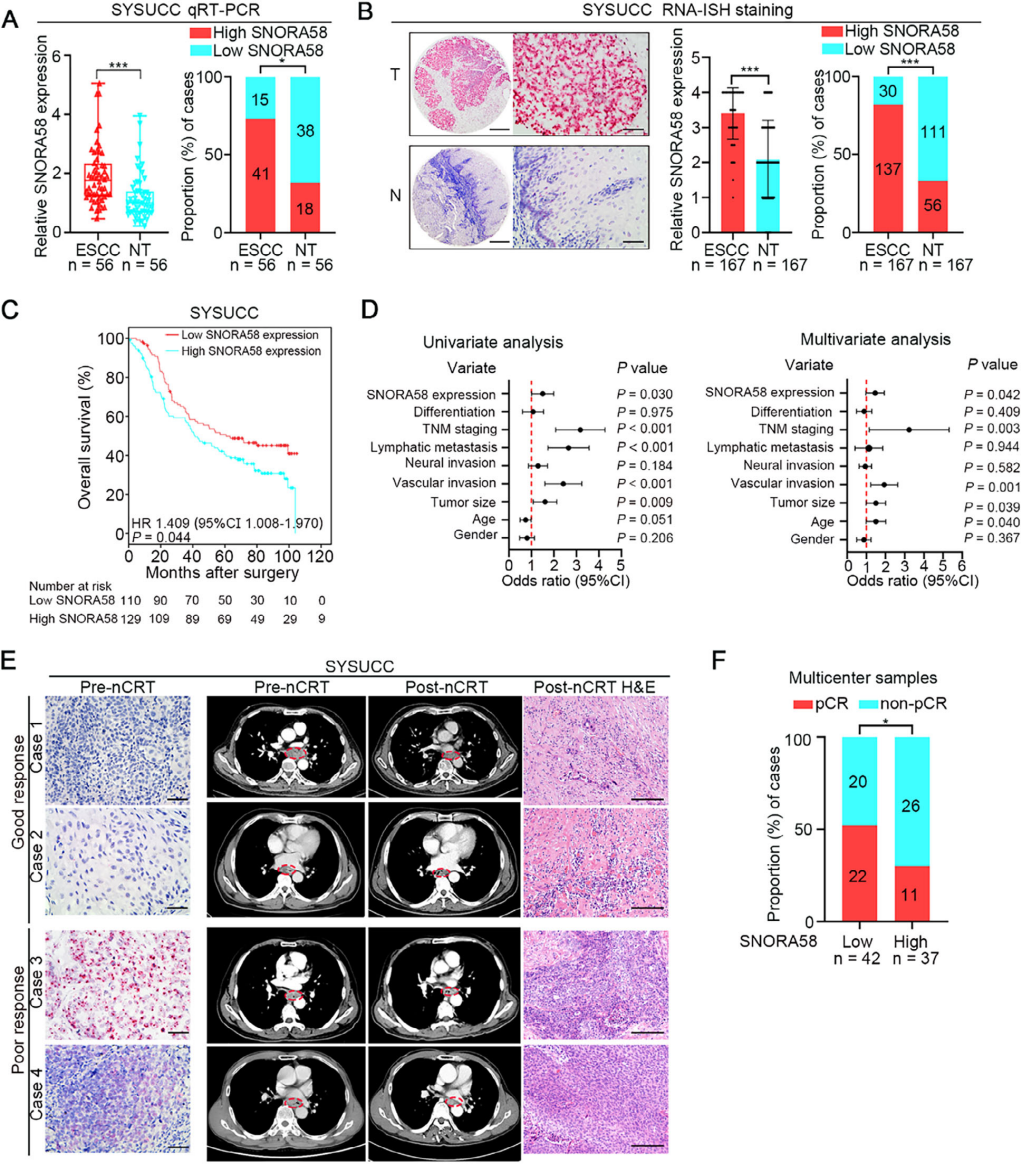
Aberrant SNORA58 overexpression indicates unfavorable outcomes and poor therapeutic efficacy of nCRT and in ESCC patients. A) The expression of SNORA58 in ESCC and corresponding nontumor samples determined by qRT‐PCR in SYSUCC cohort (*n* = 56). B) Representative images of SNORA58 expression, the expression of SNORA58 in ESCC and corresponding nontumor samples detected by RNA‐ISH staining in SYSUCC cohort (*n* = 167). Scale bar, Left: 200 µm; Right: 50 µm. C) Kaplan–Meier analysis showed the correlation between SNORA58 expression and overall survival in ESCC patients of SYSUCC cohort detected by RNA‐ISH staining (*n* = 239, *p* = 0.044). D) Univariate cox analyses (Left) and multivariate cox analyses (Right) of clinicopathological factors associated with overall survival in SYSUCC cohort detected by RNA‐ISH staining. All the bars correspond to 95% confidence intervals of hazard ratio. E) The representative images show SNORA58 staining in pre‐nCRT samples (left), computed tomography of tumors pre‐nCRT and post‐nCRT (middle) and H&E staining in post‐nCRT samples(right) of SYSUCC cohort. Scale bars, RNA‐ISH staining, 50 µm; H&E staining, 100 µm. F) The expression of SNORA58 was related to the nCRT efficacy in ESCC patients from multiple clinical centers detected by RNA‐ISH staining (*n* = 79). Data shown are analyzed by two‐tailed Student's *t*‐test (A,B), log‐rank test (C), cox regression test (D), and Chi‐square test (F), respectively. Error bars indicate mean ± SD. *p < 0.05, ***p < 0.001. ESCC, esophageal squamous cell carcinoma; NT, adjacent nontumor tissue; RNA‐ISH: RNA in situ hybridization; HR, hazard ratio; CI: confidence interval; nCRT: neoadjuvant chemoradiotherapy; pCR: pathological complete response.

To investigate the clinical significance of snoRNAs in ESCC, we conducted a snoRNA PCR array analysis using three pairs of poorly differentiated ESCC and matched nontumor tissues. SNORA58 was identified among the top 10 upregulated snoRNAs (Figure S1, Supporting Information). Quantitative validation confirmed SNORA58 overexpression in ESCC tissues compared with adjacent tissues (73.2% vs 32.1% high‐expression cases; **Figure** **1**A), which was consistent with the RNA‐ISH findings in an expanded cohort (Figure 1B; Figure S2, Supporting Information). Clinical analysis revealed significantly reduced median overall survival in high‐SNORA58 patients (43.8 vs 60.8 months; *n* = 239; Hazard Ratios (HR) = 1.409, 95% Confidence Index (CI) = 1.008–1.970; *p* = 0.044; Figure 1C). Furthermore, Univariate and multivariate Cox analysis demonstrated that high SNORA58 expression was positively associated with lymphatic metastasis as well as vascular invasion and could be an independent prognostic factor contributing to poor outcomes among ESCC patients (Figure 1D). Given the clinical importance of nCRT in advanced ESCC management, we analyzed postoperative specimens from four centers (SYSUCC, GZH‐CC, SZCC‐CAMS, and STCH). RNA in situ hybridization (RNA‐ISH) demonstrated differential therapeutic responses: low‐SNORA58 patients exhibited significant tumor regression, as evidence by Computed Tomography (CT)‐confirmed shrinkage and pathological complete response (pCR) under microscope examination, in contrast with poor outcomes in high‐SNORA58 patients (Figure 1E). Multicenter analysis revealed superior pCR rates in the low‐expression group (52.4% (22/42) vs 29.7% (11/37); Figure 1F). These findings establish SNORA58 as both a prognostic biomarker and predictor of nCRT efficacy in patients with ESCC.

### 2.2 SNORA58 Promotes Radioresistance in ESCC

**Figure 2:**
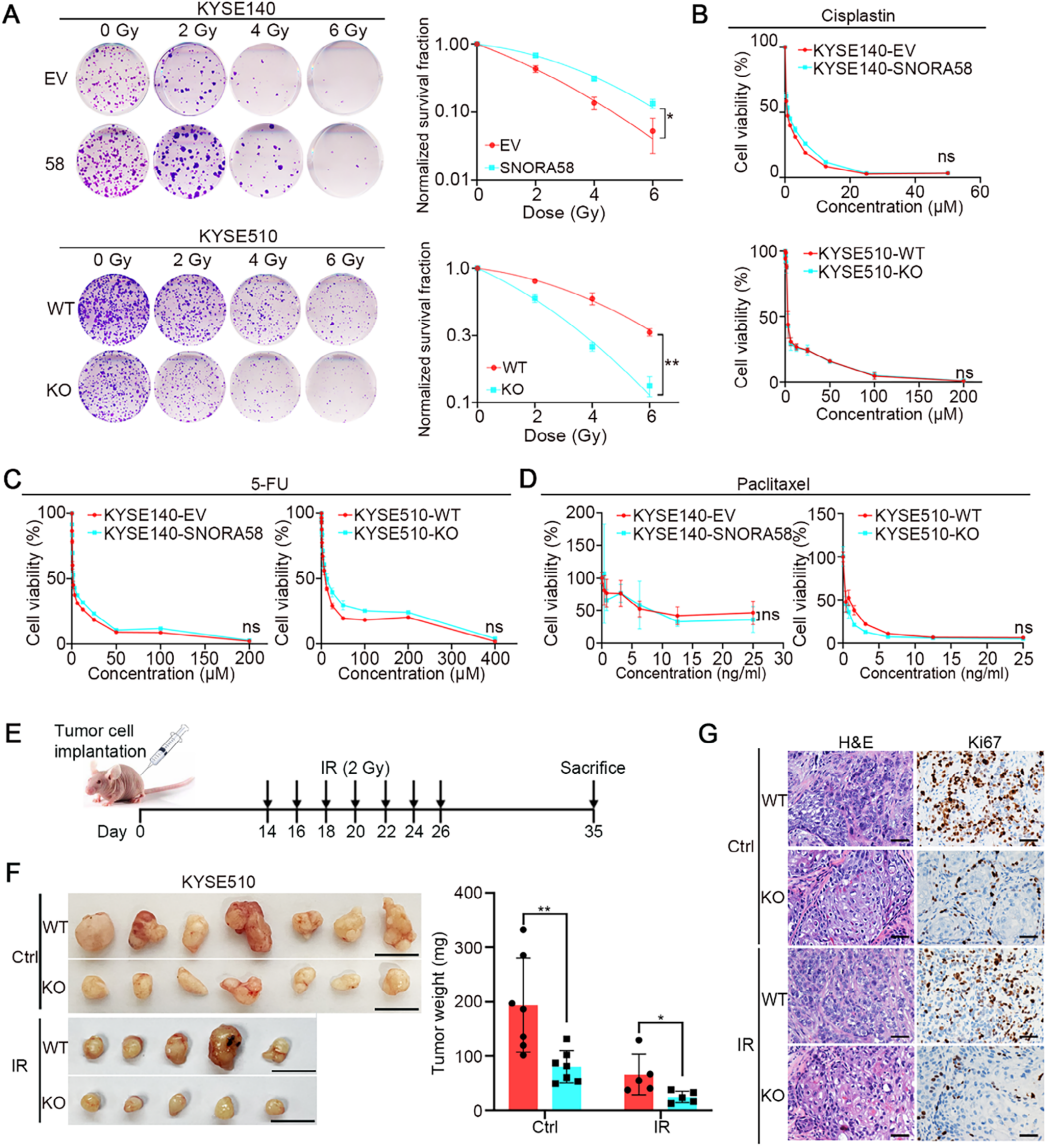
SNORA58 promotes radioresistance in ESCC. A) A clonogenic survival assay was adopted to evaluate the effect of SNORA58 on radiotherapy. B–D) Cell viability assay was applied to assess the impact of SNORA58 on chemotherapy. E) Schematic representation of the xenograft study design and experimental process. F) Xenograft mouse model derived from SNORA58 knockout and wild‐type KYSE510 cells presented the tumor sizes (left) and the tumor weights (right), respectively. Control group, *n* = 7; IR group, *n* = 5; Scale bar, 1 cm. G) The representative images of hematoxylin‐eosin (H&E) staining and Ki67 staining in tumors derived from SNORA58 knockout cells and wild‐type cells before and after IR. Scale bars, 50 µm. The data are expressed as the mean ± SD of three biological replicates and analyzed by multi‐target single‐hit model (A), two‐way ANOVA (B–D), and two‐tailed Student's *t*‐test (F), respectively. *p < 0.05, **p < 0.01. EV, empty vector; WT, wild‐type; KO, knockout; GY, gray; 5‐FU, 5‐Fluorouracil; IR, ionizing radiation; Ctrl, control; H&E, hematoxylin‐eosin; ns, nonsignificant.

To further elucidate the role of SNORA58 in nCRT, we utilized KYSE510 and KYSE180 cells with high endogenous expression of SNORA58 to generate knockout cells via the CRISPR‐Cas9 system. Conversely, KYSE140 and KYSE30 cells with low SNORA58 expression were employed to construct exogenous overexpression cells via a lentiviral system (Figure S3, Supporting Information). Clonogenic survival assays demonstrated that SNORA58 overexpression significantly reduced the radiosensitivity of ESCC cells (**Figure** **2**A, upper panel), whereas SNORA58 depletion had the opposite effect (Figure 2A, lower panel). CCK‐8 viability assays revealed no significant differences in cell viability between SNORA58‐overexpressing KYSE140 cells, SNORA58 knockout KYSE510 cells, and their respective controls following treatment with first‐line chemotherapy drugs for ESCC, including cisplatin, 5‐FU, and paclitaxel (Figure 2B–D). These findings suggest that SNORA58 does not influence ESCC chemosensitivity. To validate these in vitro findings in an in vivo setting, xenograft experiments were performed. The experimental workflow is presented in Figure 2E. As expected, compared with the control group, the SNORA58 knockout group presented declined tumor volume, tumor weight, and Ki67 expression (Figure 2F,G). Collectively, these results indicate that SNORA58 contributes to radioresistance in ESCC.

### 2.3 SNORA58 Drives Radioresistance Through SNORA58/CTCF/JNK1 Axis

**Figure 3:**
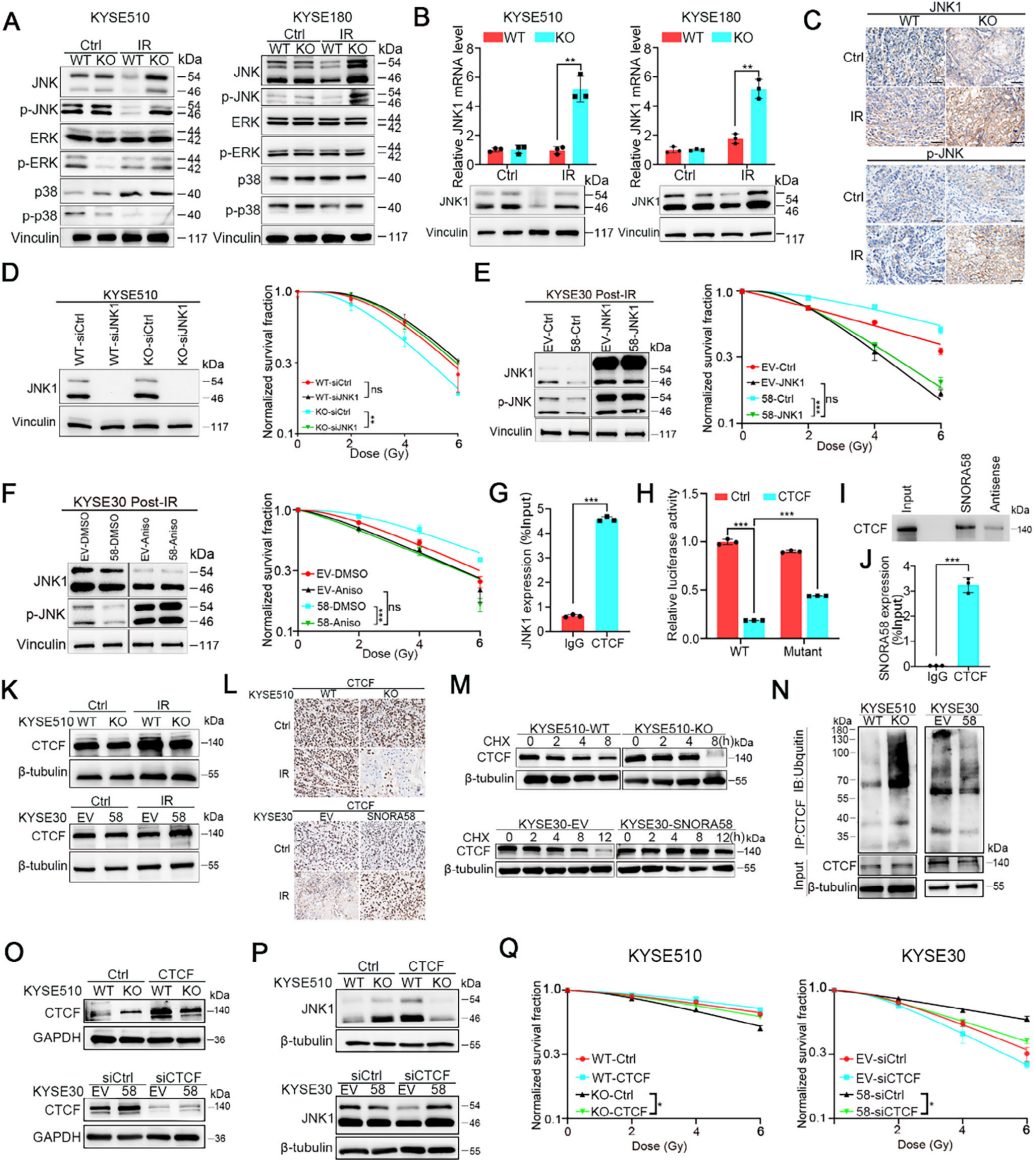
SNORA58 drives radioresistance through SNORA58/CTCF/JNK1 axis. A) Western blot assay was conducted to detect the MAPK pathway, including JNK, ERK, and p38 pathways in SNORA58 knockout cells and wild‐type cells. Vinculin served as the loading control. B) The expression of JNK1 was evaluated at RNA level by qRT‐PCR while at protein level by western blot assay in SNORA58 knockout cells and wild‐type cells. GAPDH acted as an internal control of qRT‐PCR while vinculin served as the loading control of western blot assay. C) The representative images of IHC staining of JNK1 and phosphorylated JNK in the xenograft tumor samples derived from SNORA58 knockout cells and wild‐type cells after IR. Scale bar, 50 µm. D–F) Clonogenic survival assays illustrated that JNK1 and its signaling pathway mediated SNORA58‐induced radioresistance in ESCC. G) CHIP assay validated the interaction between JNK1 and CTCF. H) Dual‐Luciferase Reporter assays indicated that CTCF is the transcriptional repressor of JNK1. I,J) RNA pulldown and RIP assays elucidated the interaction between SNORA58 and CTCF. K) Western blot assay was conducted to detect CTCF in SNORA58 knockout and overexpressing and corresponding control cells. Vinculin served as the loading control. L) Immunohistochemistry staining of CTCF in xenografts derived from SNORA58 knockout and overexpressing and corresponding control cells. Scale bars, 50 µm. M) CHX assay displayed that SNORA58 stabilized CTCF detected by Western blot post‐IR. N) Immunoprecipitation assay unraveled that SNORA58 attenuated the ubiquitination of CTCF protein post‐IR. O) Western blot assay revealed that SNORA58 stabilized the expression of CTCF after exposure to 10 Gy of IR. P) Western blot assay revealed that CTCF overexpression reversed SNORA58 knockdown‐induced JNK1 upregulation, whereas CTCF silencing counteracted SNORA58‐overexpression effects. Q) Clonogenic survival assay demonstrated that the effect of SNORA58 on radioresistance was restored by CTCF. The data are expressed as the mean ± SD of three biological replicates and analyzed by multi‐target single‐hit model (D–F,Q), two‐tailed Student's *t*‐test (B,G,H,J). ns, nonsignificant, *p < 0.05, **p < 0.01, ***p < 0.001. IR, ionizing radiation; Ctrl, control; WT, wild‐type; KO, knockout;<del user='Yun Jingping' title='Yun Jingping deleted content on: 24 Jul, 04:56 AM' class='old' id='a968813b‐67d7‐4b9a‐85f4‐2f2980ea8e98' updatedon='24 Jul, 04:56 AM' > </del>Gy, gray.

To elucidate how SNORA58 promotes ESCC radioresistance, we performed transcriptome sequencing on IR‐treated SNORA58 knockout KYSE510 and control cells. Analysis (*p* < 0.05, log2FC>1) revealed 292 downregulated and 380 upregulated differentially expressed genes (DEG) (Figure S4A, Supporting Information). GSEA revealed enrichment of the stress‐activated MAPK cascade in knockout cells (Figure S4B, Supporting Information), which is known to encompass the JNK, ERK, and p38 pathways.^[^ ^26^ ^]^ Western blot analysis confirmed increased total and phosphorylated JNK levels post‐IR in knockout cells compared with those in wild‐type cells, with unchanged ERK/p38 activity (**Figure** **3**A). qRT‒PCR screening of the JNK isoforms revealed the most pronounced JNK1 downregulation in SNORA58‐expressing KYSE510/KYSE180 cells post‐IR (Figure 3B; Figure S5, Supporting Information), indicating its suitability for subsequent downstream studies. Immunohistochemical (IHC) validation in xenografts demonstrated elevated JNK1 and p‐JNK expression in SNORA58 knockout tumors after irradiation (Figure 3C). JNK1 knockdown reversed the radiosensitization effect induced by SNORA58 knockout (Figure 3D; Figure S6A, Supporting Information). Conversely, overexpression of JNK1 in high‐SNORA58 KYSE30 cells reduced radioresistance (Figure 3E; Figure S6B, Supporting Information). Pharmacological JNK activation via anisomycin pretreatment significantly restored IR sensitivity in SNORA58‐expressing cells (Figure 3F; Figure S6C, Supporting Information). Mechanistically, SNORA58 promotes radioresistance by suppressing JNK1.

To decipher the regulatory mechanism of SNORA58 on JNK1, we hypothesized transcriptional regulation through JASPAR database analysis (https://jaspar.elixir.no/), identifying CTCF as a candidate. Five potential CTCF binding sites were identified within the JNK1 promoter region. Among these, we selected the site with the highest relative score, which is located at Chr10: 48306270–48306284 (Figure S7, Supporting Information). CHIP assays confirmed that CTCF binds to the JNK1 promoter (Figure 3G). Luciferase reporter assays demonstrated that CTCF‐mediated JNK1 suppression was abolished by binding site mutation (Figure 3H). Moreover, RNA pull‐down and RNA immunoprecipitation (RIP) experiments verified the SNORA58‐CTCF interaction (Figure 3I,J). SNORA58 knockout reduced and SNORA58 overexpression increased CTCF levels, as validated in vitro and in xenografts (Figure 3K,L). Protein stability assays further demonstrated that SNORA58 contributes to the maintenance of CTCF stability by reducing its degree of ubiquitination (Figure 3M,N; Figure S8, Supporting Information). Rescue experiments revealed that CTCF overexpression reversed SNORA58 knockout‐induced JNK1 upregulation, whereas CTCF silencing counteracted the effects of SNORA58 overexpression (Figure 3O,P). Crucially, CTCF manipulation reciprocally modulated radiosensitivity. Restoring CTCF abolished SNORA58 knockout radiosensitization, whereas depleting CTCF mitigated SNORA58‐driven radioresistance (Figure 3Q). These findings establish that SNORA58 stabilizes CTCF to repress JNK1, driving ESCC radioresistance.

### 2.4 SNORA58 Suppresses IR‐Induced Ferroptosis via JNK1‐Dependent Iron Dysregulation

**Figure 4:**
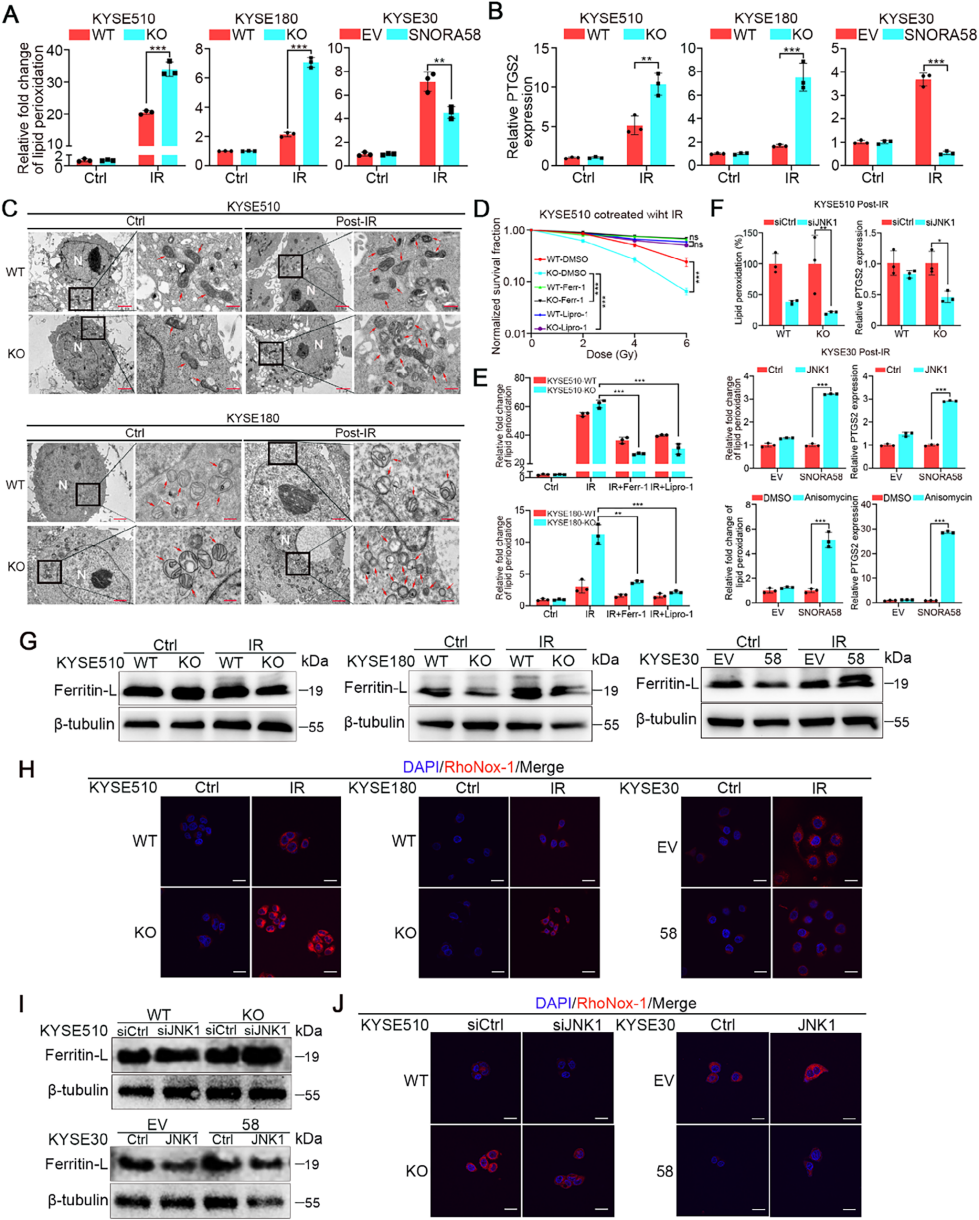
SNORA58 suppresses IR‐induced ferroptosis via JNK1‐dependent iron dysregulation. A) Lipid peroxidation was measured by C11‐BODIPY staining using flow cytometry in SNORA58 knockout, overexpressing, and the corresponding control cells after exposure to 10 Gy of IR. B) qRT‐PCR analysis of PTGS2 expression in SNORA58 knockout, overexpressing, and the corresponding control cells after exposure to 10 Gy of IR. GAPDH served as an internal control. C) Transmission electron microscopy (TEM) showed the morphological alteration of mitochondria in the indicated cells with depletion of SNORA58 after exposure to 10 Gy of IR. N, nucleus; red arrows, mitochondria; Scale bars: left (Ctrl), 2 µm; right (Post‐IR), 500 nm. D) Clonogenic survival assay in the indicated cells that were pretreated with 6 µm ferrostatin‐1, 8 µm liproxstatin‐1 or DMSO for 24 h followed by exposure to 10 Gy of IR. The survival data were normalized to those of unirradiated control cells. E) Lipid peroxidation was measured by C11‐BODIPY staining using flow cytometry in the indicated cells that were pretreated with 6 µm ferrostatin‐1, 8 µm liproxstatin‐1, or DMSO for 24 h followed by exposure to 10 Gy of IR. F)The lipid peroxidation assay illustrated that JNK1 and its signaling pathway mediated SNORA58‐regulated ferroptosis induced by IR. G) Western blot analysis revealed the positive regulation of SNORA58 on Ferritin L expression following exposure to 10 Gy of IR. H) The representative images of RHONOX‐1 staining by immunofluorescence in SNORA58 overexpressing, knockout and corresponding control cells after exposure to 10 Gy of IR.Scale bars, 20 µm. I,J) Western blot assay and RHONOX‐1 staining by immunofluorescence revealed that the role of SNORA58on Ferritin L and intracellular iron level were, reversed by JNK1 manipulation post‐IR. Scale bars, 20 µm. The data are presented as the mean ± SD of three biological replicates and analyzed by two‐tailed Student's *t*‐test (A,B,E,F) and multi‐target single‐hit model (D). *p < 0.05, **p < 0.01, ***p < 0.001. Ctrl, control; IR, ionizing radiation; WT, wild‐type; KO, knockout; EV, empty vector; Ferr‐1, ferrostatin‐1; Lipro‐1, liproxstatin‐1; Gy, gray; ns, nonsignificant.

Given that radiation‐induced apoptosis is the canonical cell death pathway,^[^ ^27^ ^]^ we assessed DNA damage (pH2AX) and apoptosis (cleaved caspase‐3) in SNORA58 knockout versus wild‐type cells. No significant intergroup differences were observed post‐IR (Figure S9A–C, Supporting Information). Screening with cell death inhibitors (ferrostatin‐1/Z‐VAD/NEC‐1/DSF/3‐MA) revealed ferroptosis as the predominant SNORA58‐regulated death modality through colony survival assays (Figure S8D–H, Supporting Information). Mechanistic validation revealed that SNORA58 knockout increased lipid peroxidation (C11‐BODIPY), PTGS2 expression, and mitochondrial shrinkage, whereas SNORA58 overexpression suppressed these ferroptosis markers (**Figure** **4**A–C). Ferrostatin‐1/liproxstatin‐1 abolished SNORA58 knockout‐induced radiosensitization and lipid peroxidation (Figure 4D,E; Figure S10A, Supporting Information). IHC staining confirmed elevated 4‐HNE (ferroptosis biomarker) in irradiated SNORA58 knockout xenograft tumors without pH2AX/cleaved caspase‐3 changes (Figure S10B, Supporting Information). JNK1 dependency was further confirmed, as SNORA58 knockout cells with JNK1 silencing exhibited reduced levels of lipid peroxidation and PTGS2 expression, whereas JNK1 overexpression or activation was demonstrated to reverse the SNORA58‐mediated suppression of ferroptosis (Figure 4F). Mechanistically, previous studies have demonstrated that JNK1 activation and its related signaling pathway can lead to an increase in intracellular Fe^2^⁺ levels by downregulating ferritin light chain (ferritin L) expression.^[^ ^28^, ^29^ ^]^ In line with these findings, SNORA58 knockout decreased ferritin L and elevated Fe^2^⁺ (RhoNox‐1), and these effects were reversible through JNK1 manipulation post‐IR (Figure 4G–J; Figure S11, Supporting Information). A screen of known ferroptosis regulators (ACSL4/GPX4/NCOA4/FTH1/DHODH/NRF2/SLC7A11) revealed no differential expression (Figure S12, Supporting Information). Collectively, these data establish that SNORA58 inhibits radiation‐induced ferroptosis primarily through disruption of JNK1‐mediated iron homeostasis.

### 2.5 JNK Activation by Anisomycin Overcomes Radioresistance In Vivo

**Figure 5:**
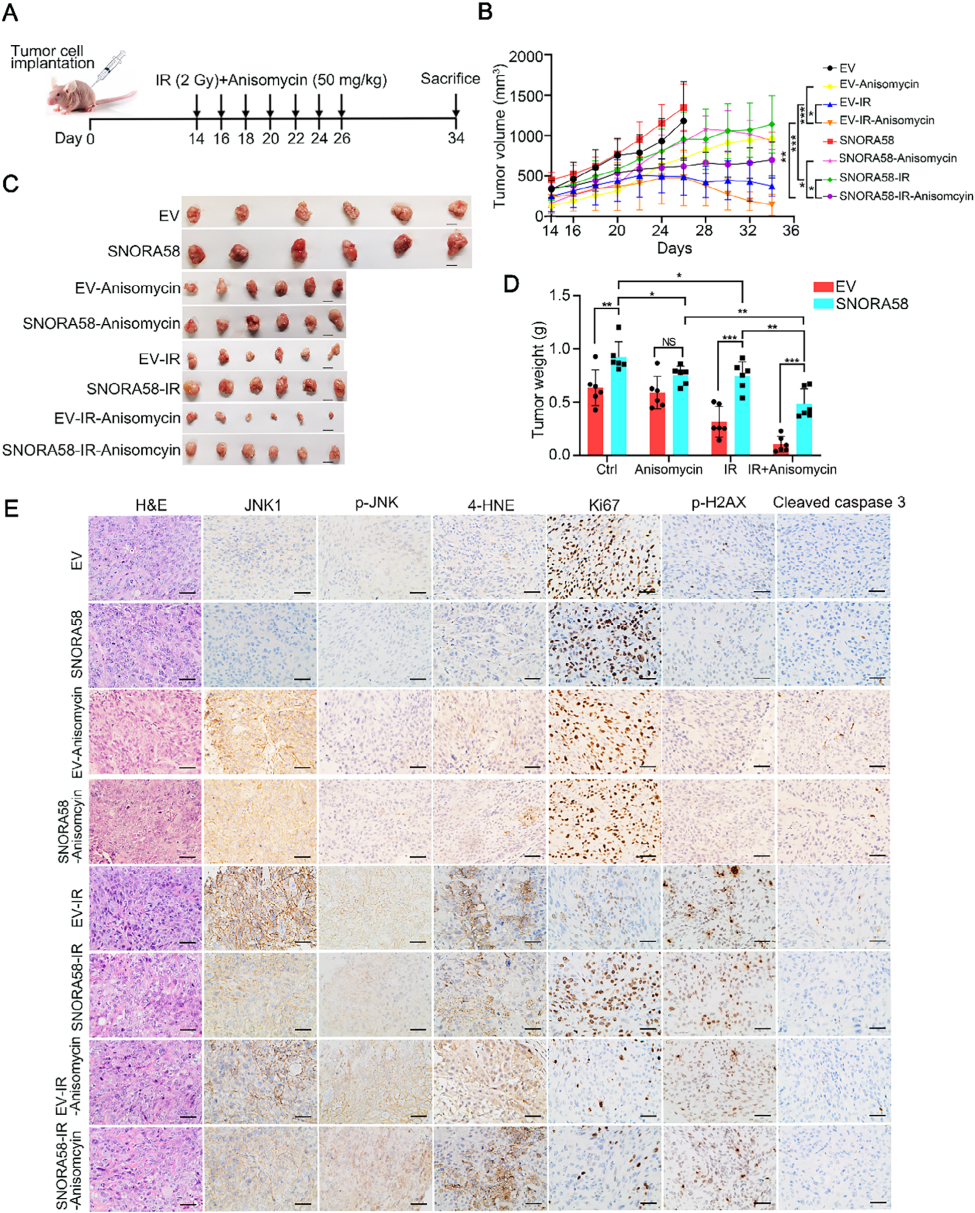
JNK activation by anisomycin overcomes radioresistance in vivo. A) Schematic diagram of xenograft mouse model derived from SNORA58‐expressing and control KYSE30 cells with indicated treatments. B) Tumor growth curves of the xenograft tumors in the indicated groups are presented (*n* = 6 for each group). C,D) Tumor sizes (C) and weights (D) in the indicated groups were exhibited. E) Representative images of H&E and IHC staining (JNK1, phospho‐JNK, 4‐HNE, Ki67, phospho‐H2AX, and cleaved caspase 3) of xenograft tumors in indicated groups. Scale bars, 50 µm. Data shown are presented as the mean ± SD and analyzed by two‐tailed Student's *t*‐test (B,D). * *p* < 0.05, ** *p* < 0.01, *** *p* < 0.001. IR, ionizing radiation; EV, empty vector; Gy, gray; ns, nonsignificant.

To validate the therapeutic potential of JNK signaling, we established KYSE30 xenograft models with SNORA58 overexpression and administered anisomycin and/or IR (**Figure** **5**A). Compared with control tumors, SNORA58‐overexpressing tumors exhibited accelerated post‐IR growth (Figure 5B). While anisomycin monotherapy showed comparable efficacy across groups (*p* = ns), anisomycin/IR combination therapy significantly reduced SNORA58‐driven tumor growth, size, and weight compared with single treatments (Figure 5B–D). IHC analysis revealed SNORA58‐mediated suppression of JNK1, p‐JNK, and 4‐HNE, with an elevated proliferation index of Ki67 post‐IR (Figure 5E). Combination therapy restored p‐JNK and 4‐HNE levels while suppressing Ki67 without altering JNK1 expression or the expression of DNA damage/apoptosis markers (pH2AX/cleaved caspase‐3, Figure 5E). Moreover, the comprehensive toxicity assessments (body weight, serum biochemistry, organ weight ratio, and organ histopathology) were performed and revealed no significant anisomycin‐related adverse effects (Figure S13, Supporting Information). Taken together, these findings confirm the ability of anisomycin to safely reverse SNORA58‐mediated radioresistance through JNK pathway activation, demonstrating its preclinical feasibility for personalized radiotherapy.

### 2.6 Clinical Prognostic Significance of SNORA58/JNK1/Ferroptosis Regulatory Axis in Post‐nCRT ESCC Patients

**Figure 6:**
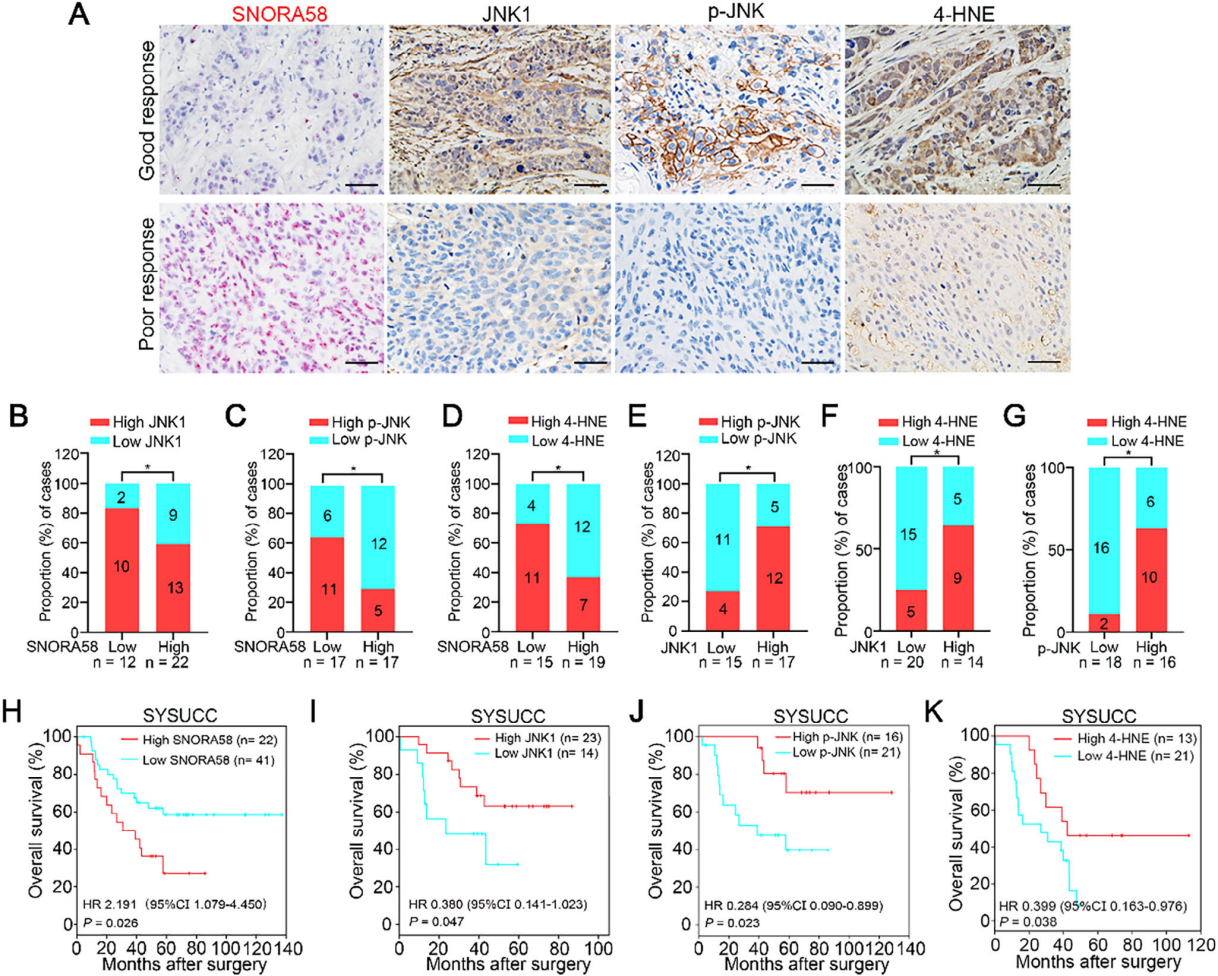
Clinical prognostic significance of SNORA58/JNK1/ferroptosis regulatory axis in post‐nCRT ESCC patients. A) Representative images of RNA‐ISH staining of SNORA58 and IHC staining of JNK1, p‐JNK, and 4‐HNE in clinical samples of good or poor response to nCRT. B–G) The correlation analysis unraveled the negative correlation between SNORA58 and JNK1 (B, *n* = 34), p‐JNK (C, *n* = 34) and 4‐HNE (D, *n* = 34), which also revealed the positive correlation between JNK1 and p‐JNK (E, *n* = 32), JNK1 and 4‐HNE (F, *n* = 34) as well as p‐JNK and 4‐HNE (G, *n* = 34). H–K) Kaplan–Meier curve analysis showed the correlation between SNORA58 (H, *n* = 63, *p* = 0.026), JNK1 (I, *n* = 37, *p* = 0.047), p‐JNK (J, *n* = 37, *p* = 0.023) and 4‐HNE (K, *n* = 34, *p* = 0.038) expression and overall survival of ESCC patients in post‐nCRT tumor specimens. Scale bars, 50 µm. *p < 0.05.

In vivo and in vitro experiments confirmed that SNORA58 inhibits JNK1‐mediated IR‐induced ferroptosis. To explore the clinical relevance of these molecules, we analyzed their expression levels in post‐nCRT ESCC samples via RNA‐ISH (SNORA58) and IHC (JNK1, p‐JNK, and 4‐HNE). Correlation and prognostic analyses were conducted to assess their clinical significance. As shown in **Figure** **6**A, good responders presented low SNORA58 and high JNK1/p‐JNK/4‐HNE expression, whereas poor responders exhibited opposite trends. Correlation analysis revealed negative associations between SNORA58 and JNK1/p‐JNK/4‐HNE (Figure 6B–D). Conversely, positive correlations were observed between SNORA58 and both JNK1 and p‐JNK and between 4‐HNE and both JNK1 and p‐JNK (Figure 6E–G). Kaplan–Meier survival analysis revealed shorter survival times in patients with high SNORA58 expression (Figure 3H, HR = 2.191, 95% CI = 1.079–4.450, log‐rank *p* = 0.026). In contrast, high JNK1, p‐JNK, or 4‐HNE expression correlated with prolonged survival and favorable prognosis (Figure 3I–K: HR = 0.380, 95% CI = 0.141–1.023, log‐rank *P* = 0.047; HR = 0.284, 95% CI = 0.090–0.899, log‐rank *p* = 0.023; and HR = 0.399, 95% CI = 0.163–0.976, log‐rank *p* = 0.038, respectively).

**Figure 7:**
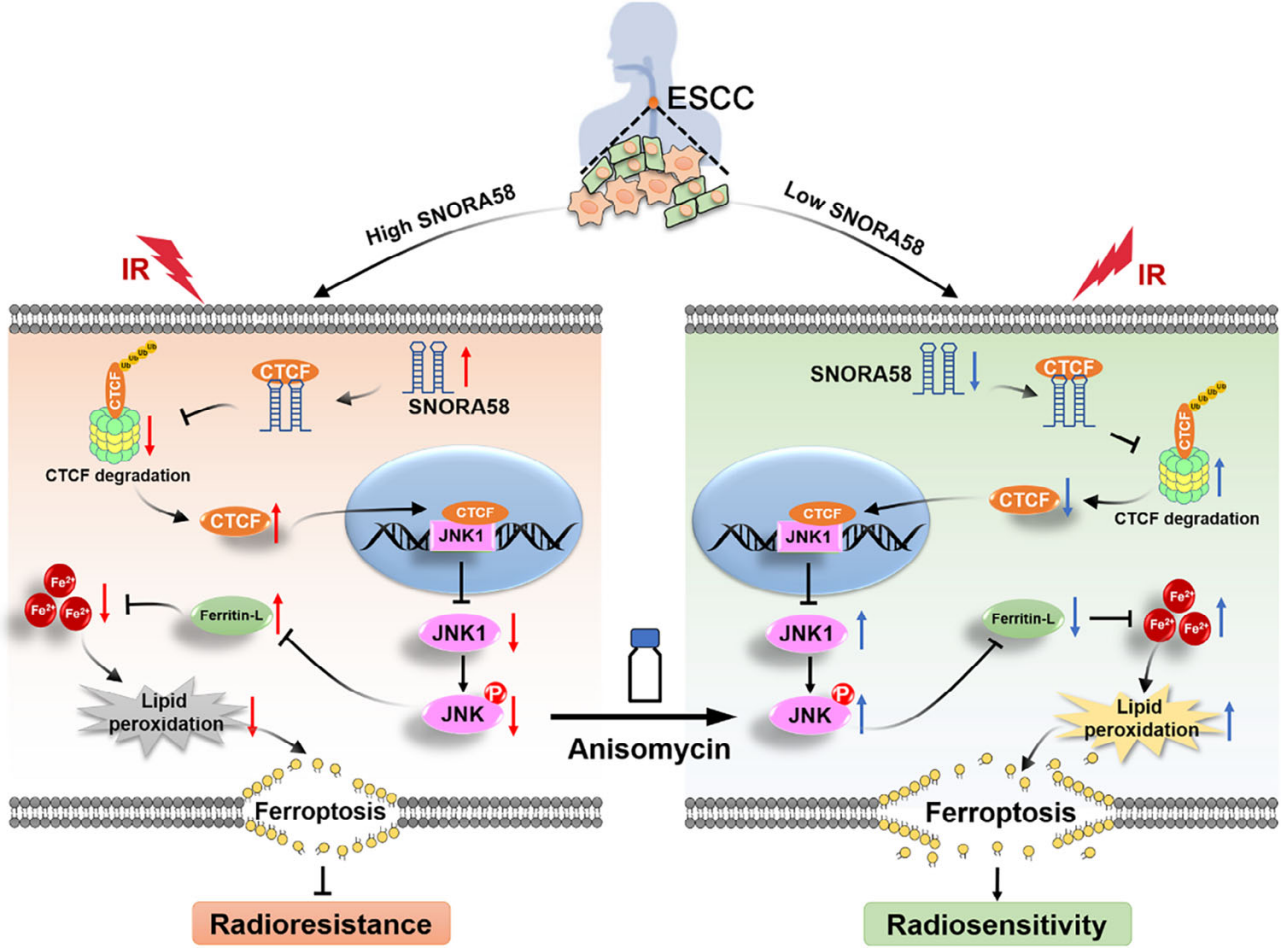
The schematic summary of the molecular mechanisms of SNORA58 facilitating radioresistance and anisomycin as a radiosensitizer in ESCC. In ESCC patients, SNORA58 exhibits heterogeneous expression patterns. Its aberrant overexpression stabilizes CTCF by inhibiting ubiquitin‐mediated degradation, leading to the attenuation of JNK1‐mediated ferroptosis induced by radiotherapy through disruption of intracellular iron homeostasis, and ultimately contributes to radioresistance. Administration of the JNK signaling pathway activator could effectively overcome SNORA58‐induced resistance.

## 3 Discussion

ESCC is a subtype of cancer that is sensitive to radiotherapy. The primary treatment strategy for managing unresectable or locally advanced ESCC involves radiotherapy alone or in combination with chemotherapy followed by surgery.^[^ ^30^ ^]^ However, the response to radiotherapy varies significantly among patients, which has a substantial impact on their prognosis. Consequently, it is crucial to identify additional molecular markers that can aid in predicting and screening for varying responses to radiotherapy in ESCC patients. This approach will facilitate the identification of patients who are resistant to radiotherapy and enable the development of individualized treatment strategies to improve therapeutic outcomes.

SnoRNAs, a class of noncoding RNAs predominantly localized in the nucleolus, can persist in body fluids and serve as potential therapeutic targets, making them promising biomarkers for cancer radiotherapy, including ESCC. However, to our knowledge, there is currently limited research on the associations between snoRNAs and ESCC radiosensitivity. In this study, we identified an upregulated snoRNA, SNORA58, in ESCC through PCR array analysis of snoRNAs, which was further validated in a large tissue microarray. Additionally, we assessed SNORA58 expression levels in plasma samples from ESCC patients and healthy controls and observed significantly elevated SNORA58 expression in the ESCC group (Figure S14, Supporting Information). Additionally, to investigate the tissue specificity of SNORA58, we further examined its expression in a multi‐tissue microarray, including renal, hepatic, colorectal, lung, and pancreatic tissues. We observed that SNORA58 is expressed across all aforementioned normal tissues and exhibits significantly elevated expression levels in the corresponding tumor tissues (Figure S15, Supporting Information). These findings indicate that SNORA58 is not restricted to esophageal tissue‐specific expression and may potentially serve as a multi‐cancer biomarker. Further validation of this conclusion will be conducted through studies incorporating a larger cohort in future research.

Furthermore, our findings indicate that high SNORA58 expression is correlated with the therapeutic efficacy of nCRT in ESCC patients based on multicenter clinical data. As reported in a previous study, the postoperative pCR rate following nCRT was ≈43.2% in ESCC patients.^[^ ^30^ ^]^ Interestingly, our results demonstrate that incorporating SNORA58 expression levels into the evaluation of postoperative pCR rates across multiple clinical centers revealed a pCR rate of only 29.7% in the high‐expression group, compared with 52.4% in the low‐expression group. Moreover, patients with high SNORA58 expression exhibited significantly shorter overall survival than those with low SNORA58 expression, with a median OS of only 30.7 months. These data suggest that SNORA58 can be used to stratify the outcomes of ESCC patients who have undergone surgery after nCRT. Functionally, we demonstrated that SNORA58 primarily reduces the sensitivity of ESCC cells to radiotherapy rather than to chemotherapy. Our findings provide the first evidence suggesting that SNORA58 may serve as a promising biomarker for predicting the therapeutic efficacy of radiotherapy in ESCC patients; this highlights the importance for clinicians to prioritize personalized treatment strategies for patients with elevated SNORA58 expression to optimize radiotherapeutic efficacy and improve clinical outcomes.

Ferroptosis has recently emerged as a novel form of radiation‐induced programmed cell death.^[^ ^22^, ^23^, ^24^ ^]^ However, the functional role of snoRNAs in linking ferroptosis with radioresistance remains unclear. In this study, our data revealed that SNORA58 alleviates radiotherapy‐induced ferroptosis by disrupting intracellular iron homeostasis via the SNORA58/CTCF/JNK1 regulatory axis. CTCF, a structural protein with RNA‐binding domains, plays a critical role in chromatin organization and gene regulation within mammalian cells.^[^ ^31^, ^32^ ^]^ In recent years, the interaction between noncoding RNAs (ncRNAs) and CTCF has emerged as a research focus. For example, hSATII RNA activates inflammatory genes by disrupting the DNA‐binding activity of CTCF,^[^ ^33^ ^]^ whereas CCAT1‐L lncRNA specifically interacts with CTCF to modulate MYC gene transcription.^[^ ^34^ ^]^ However, the relationship between snoRNAs and CTCF remains largely unexplored. This study demonstrated that SNORA58 directly binds to CTCF, stabilizing its expression by inhibiting its ubiquitin‐mediated degradation. Moreover, this study identified CTCF as a novel transcriptional repressor of JNK1. The transcriptional regulation of the JNK signaling pathway has long been a challenging area of research. While prior studies have shown that JNK regulates gene expression via interactions with the transcription factor c‐Jun, limited information exists regarding the transcriptional regulation of JNK itself.^[^ ^35^, ^36^ ^]^ Here, we report for the first time that CTCF suppresses JNK1 transcription, offering new insights into the regulatory mechanisms of the JNK and its signaling pathway. With respect to the mechanism through which JNK1 modulates radiotherapy‐induced ferroptosis, prior research has demonstrated that the activation of JNK1 and its associated signaling pathway can increase intracellular Fe^2^⁺ levels by suppressing the expression of the light chain of ferritin.^[^ ^28^, ^29^ ^]^ Regarding the activation mechanism of JNK signaling post‐IR, we hypothesize that its potential upstream kinases, such as MKK4/MKK7 or SEK1,^[^ ^28^, ^37^ ^]^ may be activated by IR exposure, subsequently initiating downstream signaling cascades. The relevant experiments will be systematically conducted to validate this hypothesis in our future research. Collectively, this study provided substantial evidence supporting the role of the JNK1‐regulatory mechanism in ferroptosis.

To further explore the radiotherapy sensitization strategy for ESCC patients with SNORA58 hyperexpression, we established a xenograft mouse model using ESCC cells overexpressing SNORA58 and subjected them to various treatments, including monotherapy with either anisomycin or radiotherapy, and their combination. Our results demonstrated that the combination therapy significantly inhibited tumor growth induced by SNORA58 hyperexpression, leading to a substantial reduction in both tumor size and weight compared with those in the groups treated with monotherapy. Intriguingly, no significant anisomycin‐associated toxicity were observed. Mechanistically, anisomycin, a JNK signaling pathway activator, effectively reversed SNORA58‐mediated suppression of the JNK signaling pathway, thereby increasing cellular ferroptosis levels and restoring the radiosensitivity of ESCC cells. Our findings provide preclinical evidence supporting the potential translational application of JNK activators to enhance radiotherapeutic efficacy with high safety in clinical settings for patients exhibiting high‐SNORA58 expression.

Our study highlights the translational potential of SNORA58 in ESCC radiotherapy. However, several limitations should be acknowledged. First, the mechanism by which SNORA58 attenuates ubiquitination‐mediated protein degradation of CTCF remains to be elucidated. Based on the results of RNA pull‐down combined with mass spectrometry analysis, ubiquitin‐associated protein 2 (UBAP2) was identified as a potential interacting partner. UBAP2 has been reported to facilitate the ubiquitination of PAICS in melanoma and SLC27A5 in hepatocellular carcinoma.^[^ ^38^, ^39^ ^]^ We hypothesize that SNORA58 may function as a guide RNA to recruit UBAP2 to CTCF, thereby enhancing CTCF ubiquitination. Future experiments will be systematically designed to validate this hypothesis. Second, since SNORA58 can be detected in plasma, future studies will explore whether exosomal mechanisms underlie its role in radioresistance.^[^ ^40^ ^]^ Third, with the continuous advancement and refinement of drug delivery systems,^[^ ^41^, ^42^ ^]^ the optimization of these systems to enhance their efficacy while minimizing toxicity continues to be a critical area for investigation. Finally, from a clinical translation perspective, the predictive value of SNORA58 for the nCRT response requires validation in multicenter prospective trials with larger cohorts. Additionally, integrating JNK activators into treatment strategies for high‐SNORA58 ESCC patients requires confirmation through clinical trials.

## 4 Conclusion

In summary, we identified SNORA58 as a signature for predicting radioresistance and elucidated its critical role in the radioresistance of ESCC. Our study reveals a previously unrecognized connection between snoRNAs and ferroptosis in radioresistance, demonstrating that SNORA58 enhances radioresistance by attenuating IR‐induced ferroptosis through the SNORA58/CTCF/JNK1 regulatory axis (**Figure** **7**). Targeting the JNK signaling pathway may provide feasible treatment strategies to improve radiotherapeutic efficacy in ESCC patients with high SNORA58 expression.

## 5 Experimental Section

### Clinical Samples and Cell Lines

Fifty‐six pairs of fresh esophageal squamous cell carcinoma (ESCC) and corresponding adjacent non‐tumor tissues were collected immediately after surgical resection at Sun Yat‐sen University Cancer Center (SYSUCC, Guangzhou, China). A human snoRNA PCR array (Cat# AS‐NR‐003, Arraystar, Rockville, USA) was employed to investigate differentially expressed snoRNAs between three pairs of fresh low‐differentiated ESCC specimens and adjacent non‐tumor tissues obtained from SYSUCC. A tissue microarray (TMA) containing 239 ESCC samples and corresponding non‐tumor tissue samples was constructed using paraffin‐embedded tissues acquired from SYSUCC. Pre‐neoadjuvant chemoradiotherapy (nCRT) and post‐nCRT ESCC samples were collected from multiple clinical centers, including Sun Yat‐sen University Cancer Center (38 cases), Affiliated Cancer Hospital and Institute of Guangzhou Medical University (9 cases), Shenzhen Center of Cancer Hospital Chinese Academy of Medical Sciences (15 cases), and Shantou Central Hospital (17 cases). The use of all clinical samples in this study was approved by the relevant Committees for Ethical Review of Research. The human ESCC cell lines used in this study, including KYSE30, KYSE140, KYSE510, KYSE180, KYSE410, KYSE150, KYSE520, HK, and TE1, were procured and authenticated from DSMZ (Braunschweig, Germany). All these cell lines were routinely cultured in RPMI 1640 medium (GibcoBRL, Grand Island, NY) supplemented with 10% fetal bovine serum (FBS, GibcoBRL, Grand Island, NY) and 1% penicillin/streptomycin at 37 °C in a humidified incubator with 5% CO_2_. All cell lines were identified by STR profiling and tested for mycoplasma contamination using MycoAlert (Lonza).

### RNA Isolation and Quantitative Real‐Time PCR Analysis

Total RNA was extracted from tissues and cells using RNAiso Plus reagent (TaKaRa, Japan) in accordance with the manufacturer's instructions. For plasma samples obtained from ESCC patients or healthy volunteers, total RNA extraction was performed using the miRNeasy Serum/Plasma Kit (Qiagen, Germany) following the manufacturer's protocol. Complementary DNA synthesis was carried out using the HiScript II Q RT SuperMix Kit (Vazyme Biotech, Nanjing, China). qRT‐PCR analysis was performed as previously described, with GAPDH or 5s rRNA used as internal controls. Relative RNA expression levels were determined using the 2^−ΔΔCt^ method. Each sample was subjected to three independent replicate experiments. The primer sequences are provided in Table S1 (Supporting Information).

### RNA In Situ Hybridization Staining

The SnoRNA in situ hybridization was performed manually using the PinpoRNATM Multiplex Fluorescent RNA In Situ Hybridization Kit (GD Pinpoease Biotech Co., Ltd., Cat#PIF1000) according to the manufacturer's instructions. A series of short probes (Cat#6778361‐A1), designed based on patented algorithms (China Patent No. ZL202110581853.9), were sequentially hybridized to the SNORA58 RNA sequence, covering the region from nucleotide 1 to 180. The staining intensity was scored independently by two pathologists who were blinded to the clinical information, using a scale of 0 to 4. The scoring criteria were as follows: 0 = no staining or ≤ 1 dot per 20 cells, 1 = 1 dot/cell, 2 = 2–3 dots/cell, 3 = 4–10 dots/cell, and 4 = > 10 dots/cell.

### Transfection of Plasmids and Small‐Interfering RNA

PLVX‐SNORA58, pEV‐M02‐JNK1, and their corresponding control plasmids were obtained from GeneCopoeia (Rockville, MD). The siRNA targeting JNK1 and the matched control siRNA were provided by Genepharma (Shanghai, China). The sequence of siJNK1 was 5ʹ‐GCTCATGGATGCAAATCTT‐3ʹ. SNORA58 knockout cells were generated using the CRISPR/Cas9 genome editing system as previously described.^[^ ^43^, ^44^ ^]^ The sequence of the SNORA58 sgRNA was 5ʹ‐GCTCTAACCAGCTTCATCAG‐3ʹ. Plasmids for SNORA58 overexpression and knockout were transfected into 293FT cells using a lentiviral packaging mix (Invitrogen, Carlsbad, CA). Hilymax (Dojindo Molecular Technologies, Kumamoto, Japan) and Lipofectamine RNAiMAX (Thermofisher Scientific, Rockford, IL) were used as transfection reagents for the transient transfection of plasmids and siRNA, respectively, according to the manufacturer's instructions. The efficiency of SNORA58 overexpression or knockout was assessed by qRT‐PCR, while western blotting was used to evaluate the efficiency of JNK1 overexpression or silencing.

### Clonogenic Survival Assay

The radiosensitivity of ESCC cells was assessed using a clonogenic survival assay. Cells (1000–3000 per well) were seeded in triplicate into six‐well plates and subjected to various treatments, including irradiation (IR) and pretreatment with specific inhibitors or DMSO as controls. Irradiation was performed using the Rad Source RS2000 X‐RAD irradiation system (Rad Source, USA) at specified doses. After 8–10 days, cell colonies (>50 cells/colony) were stained with 0.5% crystal violet and quantified using ImageJ software. The survival fraction of cells was calculated using GraphPad Prism 6 and normalized to that of non‐irradiated cells. The dose‐survival curve was fitted according to the single‐hit multi‐targeted model using the formula SF = 1‐(1‐exp(−k*D))^N.

### Cell Viability Assay

The chemosensitivity of ESCC cells was evaluated using a cell viability assay. Cells (1000 per well) were seeded into 96‐well plates and treated with various chemotherapy agents in triplicate at a minimum. Cell viability was measured using the Cell Counting Kit‐8 (CCK‐8, Dojindo Molecular Technologies, Kumamoto, Japan) according to the manufacturer's instructions.

### RNA Sequencing Analysis

Total RNA was extracted from SNORA58 knockout and wild‐type KYSE510 cells after irradiation (IR) using the RNAiso Plus reagent and subjected to sequencing on the Illumina HiSeqTM 2500/4000 platform by Gene Denovo Biotechnology Co., Ltd. (Guangzhou, China). RNA purity was evaluated using the NanoDrop 2000 spectrophotometer (Thermo Fisher Scientific, Wilmington, DE), and RNA integrity was assessed using the RNA Nano 6000 assay kit on the Agilent Bioanalyzer 2100 system (Agilent Technologies, Santa Clara, CA, USA). Differential expression analysis of RNA‐seq data was conducted using DESeq2 (version 1.22.2). Furthermore, gene set enrichment analysis (GSEA) was performed using the GSEA software to investigate the regulatory signaling pathways enriched by differentially expressed genes (DEGs).

### Immunohistochemistry Staining

Immunohistochemistry staining was performed according to previously described protocols.^[^ ^45^ ^]^ Briefly, paraffin‐embedded sections were deparaffinized in xylene, rehydrated with gradient ethanol, and blocked with 3% H2O2. Antigen retrieval was carried out using citrate acid buffer. The slides were then blocked with 5% goat serum in PBS for 1 h at room temperature, followed by overnight incubation with primary antibodies (Table S2, Supporting Information) at 4 °C. Staining was visualized using the EnVision detection system (Dako, Glostrup, Denmark), and nuclei were counterstained with Meyer's hematoxylin. Images were captured using an Olympus FSX100 microscope (Olympus, Tokyo, Japan). IHC staining results were independently evaluated by two pathologists who were blinded to the patients’ clinical characteristics. The proportion of immune‐positive cells was scored on a scale from 0 to 4 (0%, 1–25%, 26–50%, 51–75%, and 76–100%). The staining intensity was scored as negative (0), weak (1), moderate (2), or strong (3). The final score was calculated as the product of the proportion score and the intensity score (ranging from 0 to 12). The median IHC score was used as the cut‐off value to determine upregulation or downregulation of the target genes, as illustrated in the corresponding figure legend.

### Chromatin Immunoprecipitation

ChIP analysis was performed on KYSE30 cells (4 × 10^6) using the Pierce Magnetic ChIP Kit (#26157, ThermoFisher) with minor protocol modifications. Cells were fixed with 1% formaldehyde (10 min, 25 °C), quenched with 0.125 M glycine, and lysed in Membrane Extraction Buffer containing protease/phosphatase inhibitors. Chromatin was digested with micrococcal nuclease (1 U/µl, 37 °C, 15 min) and fragmented to 200–500 bp using a Qsonica Q700 ultrasonicator (three 20‐s pulses, 20‐s intervals). Immunoprecipitation employed 5 µg anti‐CTCF antibody (#ab128873, Abcam) or rabbit IgG control (#2729, CST) incubated overnight at 4 °C with 500 µg chromatin. Antibody complexes were captured using protein A/G magnetic beads for 2 h at 4 °C, washed sequentially, and eluted in 1% SDS/0.1 M NaHCO₃. Crosslinks were reversed for 40 min at 65 °C, followed by proteinase K digestion (1 mg mL^−1^) for 1 h at 55 °C. Purified DNA was quantified and analyzed by qPCR (ChamQ SYBR qPCR Master Mix, #Q311‐02, Vazyme) using CTCF‐specific primers. The primer sequences are provided in Table S1 (Supporting Information).

### Dual‐Luciferase Reporter Gene Assay

Transcriptional activity was assessed using the Dual‐Luciferase Reporter Assay System (#11402ES60, Yeasen Biotechnology, China). KYSE30 cells were cultured in 24‐well plates and transfected at 60–80% confluence with combinations of 100 ng CTCF plasmids, wild‐type (WT) JNK1 promoter plasmids, or CTCF plus mutant JNK1 promoter plasmids. Additionally, 10 ng of the Renilla luciferase control vector pRL‐TK (IGEbio, China) was included in the transfection mixture using Lipofectamine™ 3000 (#L3000015, Thermo Fisher Scientific, USA). Cells were lysed 48 h post‐transfection, and luciferase activities were measured with a Spark™ 10M microplate reader (TECAN, Switzerland). Data were normalized to the Firefly/Renilla ratio to account for variability in transfection efficiency.

### RNA Immunoprecipitation Assay

RNA‐protein interactions were analyzed using the Magna RIP™ RNA‐Binding Protein Immunoprecipitation Kit (Millipore, #17‐700) following optimized protocols. KYSE30 cells overexpressing SNORA58 (2 × 10^7) were lysed in ice‐cold RIP Lysis Buffer with RNase and protease inhibitors, followed by a freeze‐thaw cycle and centrifugation. Immunoprecipitation was performed with 5 µg of anti‐CTCF antibody (#ab128873, Abcam) or rabbit IgG control (Millipore, #PP64B), incubated with the lysate at 4 °C overnight. RNA‐protein complexes were captured using protein A/G magnetic beads for 2 h at 4 °C with rotation, washed with RIP Wash Buffer, and digested with proteinase K at 55 °C for 30 min. RNA was extracted using Trizol and precipitated with isopropyl alcohol. The purified RNA was quantified and reverse‐transcribed using HiScript II Q RT SuperMix (Vazyme, #R223‐01). The enrichment of SNORA58 was quantified by qPCR employing the ΔCt method and expressed as a percentage of input relative to IgG control.

### Cycloheximide Chase Assay

Protein stability and degradation kinetics were assessed in isogenic cell models using cycloheximide (CHX) treatment. Specifically, the indicated cells were pre‐treated with 10 Gy irradiation and subsequently exposed to 20 µm cycloheximide (CHX, catalog #HY‐12320, MCE) for durations ranging from 0 to 12 h (at time points of 0, 2, 4, 6, 8, and 12 h). Thereafter, the cells were harvested at the designated time intervals, lysed, and subjected to Western blot analysis.

### Immunoprecipitation Assay

The level of protein ubiquitination was assessed using a modified immunoprecipitation protocol. Cells were pre‐treated with 10 µm MG132 (#HY‐13259, MCE) 12 h post 10 Gy irradiation. Protein concentrations were normalized to ensure equal loading prior to the immunoprecipitation assay. Subsequently, 500 µg of total protein lysates were incubated overnight at 4 °C with either 4 µg of anti‐CTCF antibody (#ab128873, Abcam) or rabbit IgG control (#2729, CST) in IP buffer. Following this incubation, the mixture was subjected to gentle rotation for 2 h at 4 °C in the presence of protein A/G agarose beads (#B23201, Selleck, USA). The immunoprecipitates were then washed and resolved by 10% SDS‐PAGE, followed by Western blot analysis using the primary antibody Ubiquitin (P4D1) (Santa Cruz, #sc‐8017; dilution 1:200).

### Immunofluorescence Staining

Immunofluorescence (IF) staining was performed as previously described.^[^ ^46^ ^]^ KYSE150 and KYSE180 cells were seeded in confocal dishes at a density of 5 × 10^4 cells per well for overnight culture and exposed to 10 Gy of irradiation (IR) the following day. After 12 h of culture, the cells were fixed with 4% paraformaldehyde and permeabilized with 0.1% Triton X‐100. Subsequently, the cells were blocked with 5% BSA for 1 h at room temperature, incubated with primary antibodies (Table S2, Supporting Information) overnight at 4 °C, and stained with secondary antibodies (Table S2, Supporting Information) the next day at room temperature. Finally, the nuclei were counterstained with DAPI, and images were captured using a confocal fluorescence microscope (Olympus FV1000, Tokyo, Japan).

### Antibodies and Western Blot Assay

The indicated cells were cultured until they reached 70–90% confluence, harvested, and lysed for 30 min on ice in RIPA buffer (Beyotime, China) supplemented with protease and phosphatase inhibitors (Roche, Basel, Switzerland). Western blot analysis was conducted according to the standard protocol. The antibodies used in this study are listed in Table S2 (Supporting Information).

### Apoptosis Assay

The cells were irradiated with or without a dose of 10 Gy and subsequently subjected to apoptosis detection using Annexin V and 7‐aminoactinomycin D (7‐AAD, 559763, BD Biosciences) according to the manufacturer's instructions. Flow cytometry was then performed for apoptosis analysis using a CytoFLEX flow cytometer (Beckman Coulter, California). The data were finally analyzed using FlowJo software (Version 10.7.2, BD Biosciences).

### Lipid Peroxidation Assay

Cells (5 × 10^4 per well) were seeded in 12‐well plates in triplicate and pretreated with or without drugs for 24 h, followed by exposure to the indicated doses of irradiation (IR). After an additional 12 h of incubation, fresh medium containing 5 µm BODIPY 581/591 C11 dye (Invitrogen, Carlsbad, CA) was added to each well and incubated at 37 °C for 30 min. The cells were then gently washed with cold PBS and analyzed by flow cytometry using a CytoFLEX flow cytometer (Beckman Coulter, California). Finally, the data were analyzed using FlowJo software (Version 10.7.2, BD Biosciences).

### Transmission Electron Microscopy

Transmission electron microscopy (TEM) was employed for ultrastructural analysis of mitochondria. Specifically, SNORA58 knockout or wild‐type KYSE510 and KYSE180 cells were treated with or without ionizing radiation (IR). After trypsin digestion, the cell pellets were harvested, washed three times with 0.1 M phosphate buffer (pH 7.2–7.4), and subsequently fixed in 1% osmium tetroxide (OsO4) for 2 h at 4 °C. The samples were then rinsed three times with 0.1 M phosphate buffer, dehydrated through a graded ethanol series, and embedded in Spurr's resin. Ultrathin sections were prepared using a LEICA EM UC7 ultramicrotome and stained with either 2% uranyl acetate or 3% lead citrate. Finally, TEM images were acquired using a JEOL JEM‐1200EX transmission electron microscope.

### Mice and Treatment

Four‐week‐old male athymic BALB/c nude mice were purchased from the Guangdong Research Center of Laboratory Animal (Guangzhou, China). SNORA58 knockout and wild‐type KYSE510 cells (5 × 10^6) were subcutaneously injected into the right flanks of the mice. After 2 weeks, when the tumor volume reached ≈150 mm^3^, the mice were randomly divided into a control group without ionizing radiation (IR, *n* = 7) and an experimental group subjected to fractionated radiation (2 Gy every 2 days for 7 days, *n* = 5), as illustrated schematically in Figure 2E. The tumors were then excised and weighed after euthanasia for subsequent IHC analysis. To investigate whether the combination of anisomycin (MCE, USA), a JNK activator, with IR could resensitize SNORA58‐overexpressing radioresistant cells to IR, SNORA58‐expressing and control KYSE30 cells (4 × 10^6) were subcutaneously injected into the right flanks of nude mice. Two weeks later, when the tumors reached an appropriate size, the mice were randomly assigned to the following groups: control group without IR (*n* = 6), anisomycin‐only group (50 mg kg^−1^, *n* = 6), IR‐only group (fractionated radiation, 2 Gy every 2 days for 7 days, *n* = 6), and combination group (fractionated radiation, 2 Gy every 2 days for 7 days, plus anisomycin at 50 mg kg^−1^, *n* = 6), as depicted in Figure 5A. Tumor size was measured every 2 days, and tumor volume was calculated using the formula: length × width^2^ × 0.5. Mice were euthanized after completing the treatment regimen. Xenograft tumors were excised, weighed, and prepared for IHC analysis. All animal experiments were conducted in accordance with guidelines approved by the SYSUCC Animal Care and Use Committee.

### Statistical Analysis

The experimental data in this study were statistically analyzed using SPSS (version 22.0, Chicago, IL) and GraphPad Prism 8.0. Differences between two groups were assessed using a two‐tailed Student's *t*‐test. For independence tests between two categorical variables, Fisher's exact test was applied when the sample size was less than 40; otherwise, the Chi‐square test was utilized. Kaplan–Meier survival curves were generated, and log‐rank tests and Cox proportional hazards models were employed for overall survival analyses. All results are expressed as mean ± SD, and statistical significance was defined as *p* < 0.05.

## Conflict of Interest

The authors declare no conflict of interest.

## Author Contributions

Y.Z., F.L., Y.H., and Y.F. contributed equally to this work. Y.L.Z., F.Y.L., Y.H.H., and Y.F.F. conducted experiments and interpretated results. Y.L.Z. wrote the manuscript. X.Y. and T.T.Z. carried out immunohistochemistry staining assays. Y.H.H. and Y.F.F. evaluated results of immunohistochemistry staining. X.Y., L.J., and X.H.L. participated in the collection of clinical specimens. J.J.W. and Y.L. provided material support. X.Y.G. aided in drafting the manuscript. Y.Y.W., C.Y.C., and J.P.Y. supervised the project.

## Supporting information



Supporting Information

## Data Availability

The data that support the findings of this study are available from the corresponding author upon reasonable request.
