# Supplementary material for: SNORA58 Facilitates Radioresistance via Suppressing JNK1‐Mediated Ferroptosis in Esophageal Squamous Cell Carcinoma
Source: Adv Sci (Weinh). 2025 Jul 26;12(40):e08515. doi: 10.1002/advs.202508515 (PMC12561220; doi:10.1002/advs.202508515)
Supplement: Supplementary file 1 — Supporting Information [file ADVS-12-e08515-s001.docx]

**Supplementary information**

**SNORA58 Facilitates Radioresistance via Suppressing JNK1-Mediated Ferroptosis in Esophageal Squamous Cell Carcinoma**

Yinli Zheng^1,2†^, Fangyi Liu^1,2†^, Yuhua Huang^1,2†^, Yanfen Feng^1,2†^, Xia Yang ^1,2^, Jinjun Wu^3^, Xin Yang^4^, Xuanhao Lin^5^, Lives Jiang^6^, Tingting Zeng^1^, Yan Li^1^, Xinyuan Guan^1,7,8^, Yuanyuan Wang^5*^, Chunyan Chen^1,9*^, Jingping Yun^1,2*^

This file contains the following supplementary materials: Figures S1 to S15 along with their respective legends, and Tables S1 to S2.

**Supplementary figures**


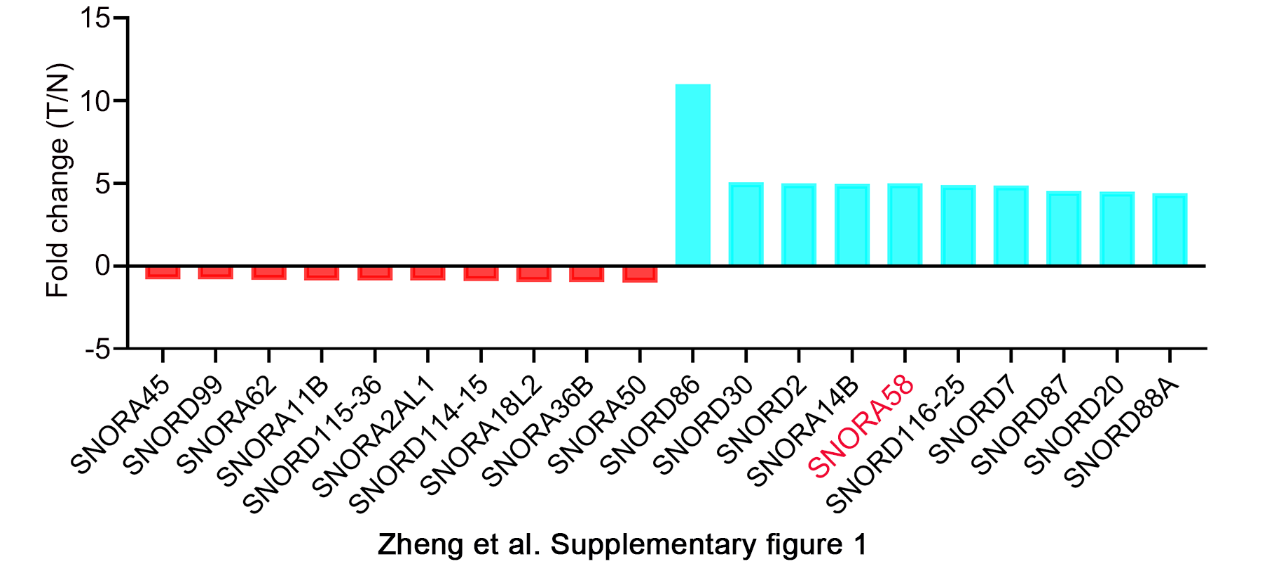


**Figure S1** The top 10 upregulated and downregulated differential snoRNAs analyzed by snoRNA PCR array in three pairs of esophageal squamous cell carcinoma and corresponding nontumor tissues.

**
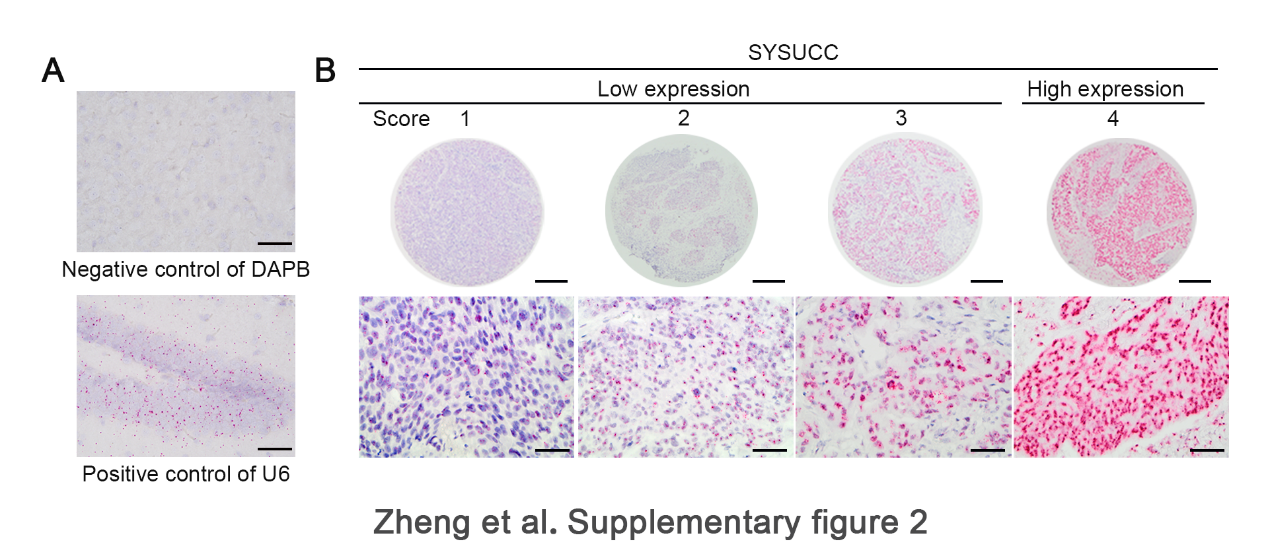
Figure S2** Representative images of positive/negative control. (**A**) and scoring criteria of SNORA58 (**B**) in ESCC TMA measured by RNA-ISH staining. Scale bar, Up: 400 µm; Down: 50 µm.


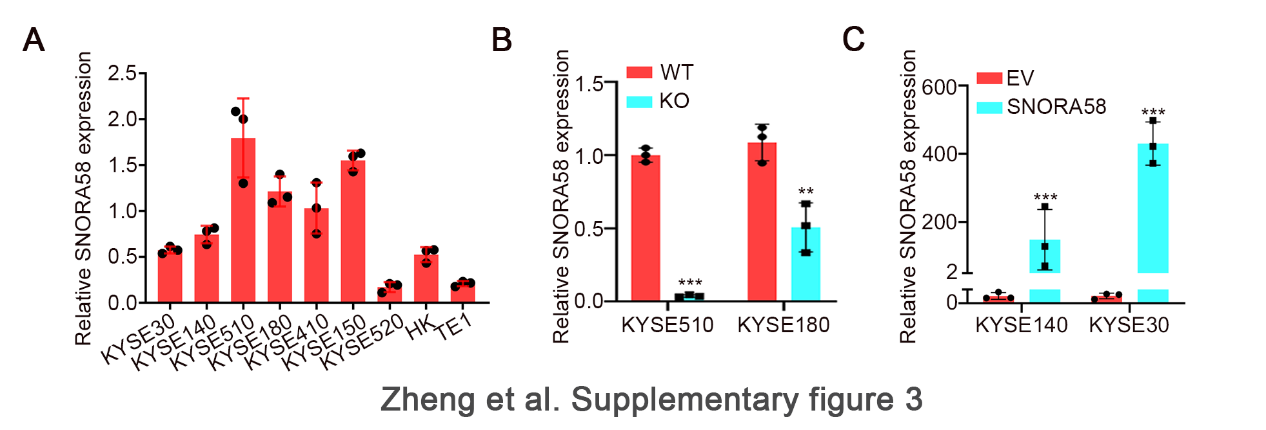


**Figure S3** The expression of SNORA58 in ESCC cells and the efficiency of ectopic overexpressed or knockout SNORA58 in indicated ESCC cells were determined by qRT-PCR. (**A**) The expression level of SNORA58 in ESCC cells was determined by qRT-PCR. (**B**) The knockout efficiency of SNORA58 in KYSE510 and KYSE180 cells was determined by qRT-PCR. (**C**) The overexpressed efficiency of SNORA58 in KYSE140 and KYSE30 cells was determined by qRT-PCR. GAPDH served as an internal control. The data are expressed as the mean ± SD of three biological replicates and analyzed by unpaired *t-test* (**B** and **C**). ***P* < 0.01, ****P* < 0.001. WT, wild-type; KO, knockout; EV, empty vector.


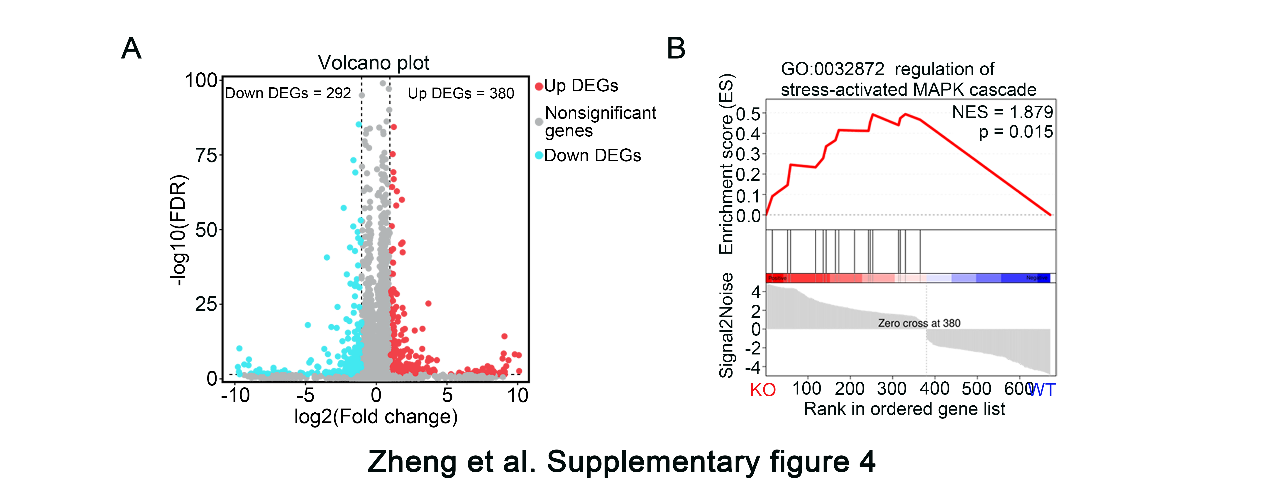


**Figure S4** Transcriptome Sequencing and Gene Set Enrichment Analysis (GSEA). (**A**) Volcano plot of differentially expressed genes (DEGs, Fold change > 2, FDR < 0.05) between SNORA58 knockout cells and wild-type cells after IR analyzed by transcriptome sequencing. (**B**) GSEA of DEGs revealed that stress-activated MAPK cascade was activated after depletion of SNORA58. NES, normalized enrichment score; FDR, false discovery rate.


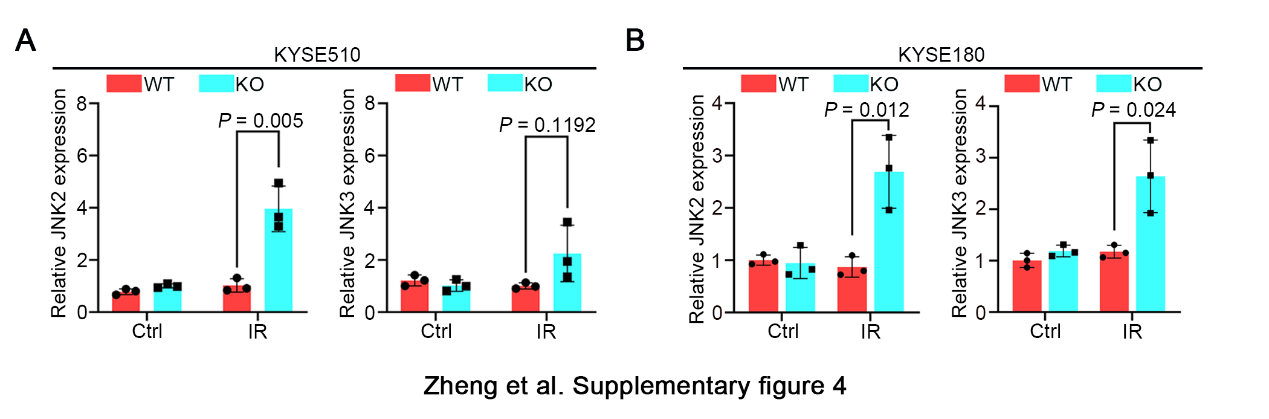


**Figure S5** The expression of JNK2 and JNK3 of SNORA58 knockout cells and wild-type cells was determined by qRT-PCR in KYSE510 cells (**A**) and KYSE180 cells (**B**). GAPDH served as an internal control. The data are expressed as the mean ± SD of three biological replicates and analyzed by unpaired t-test. Ctrl, control; IR, ionizing radiation; WT, wild-type; KO, knockout.


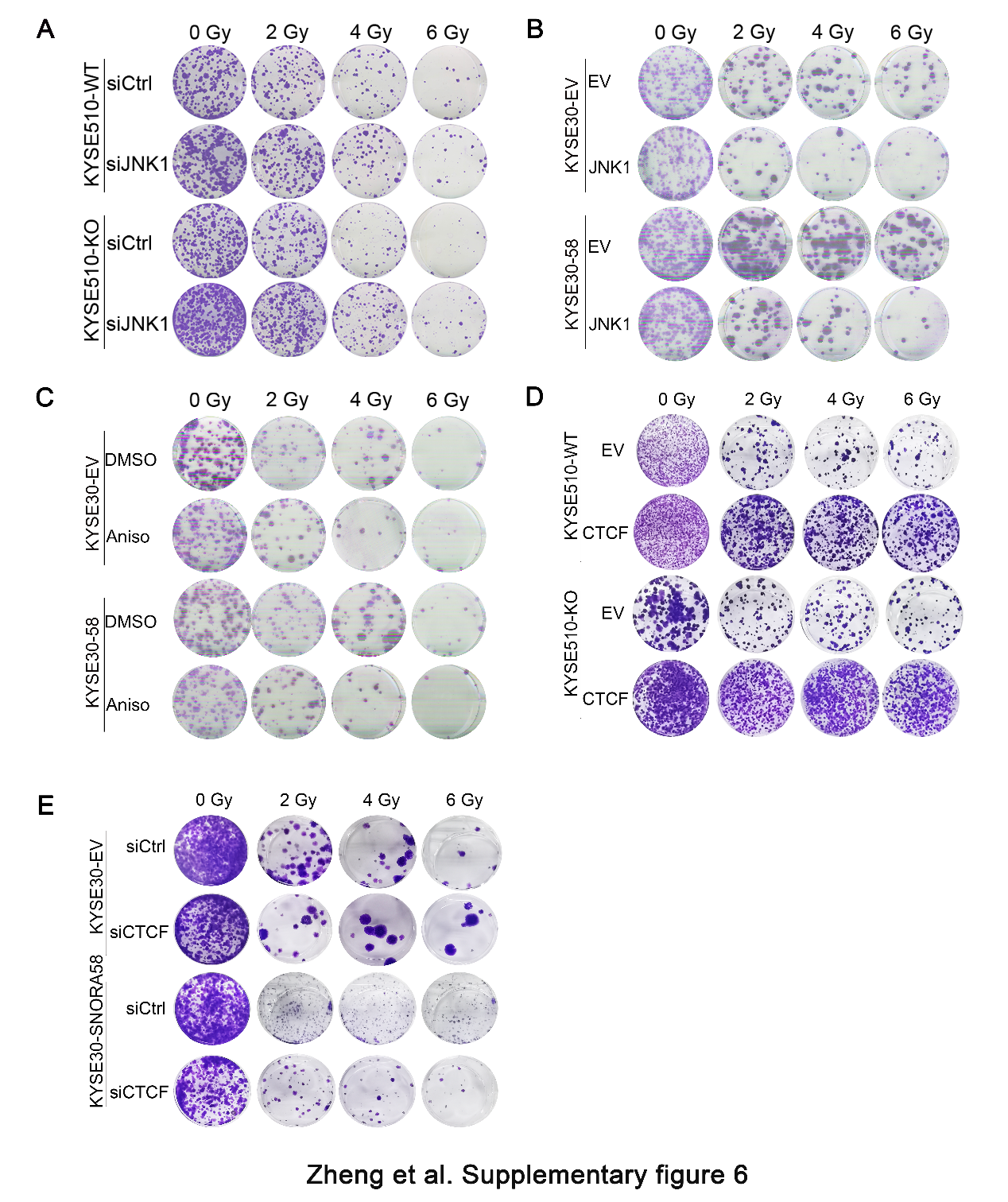


**Figure S6** Representative images of the clonogenic survival assay following gradient-dose radiotherapy in indicated cells. (**A-E**) Representative images of the clonogenic survival assay in SNORA58 knockout cells treated with JNK1 silencing (A) and CTCF overexpression (D); in SNORA58 expressing cells treated with JNK1 transfection, (B) anisomycin treatment (**C**), and CTCF silencing (**E**). Ctrl, control; WT, wild-type; KO, knockout; EV, empty vector; 58, SNORA58; Gy, gray.


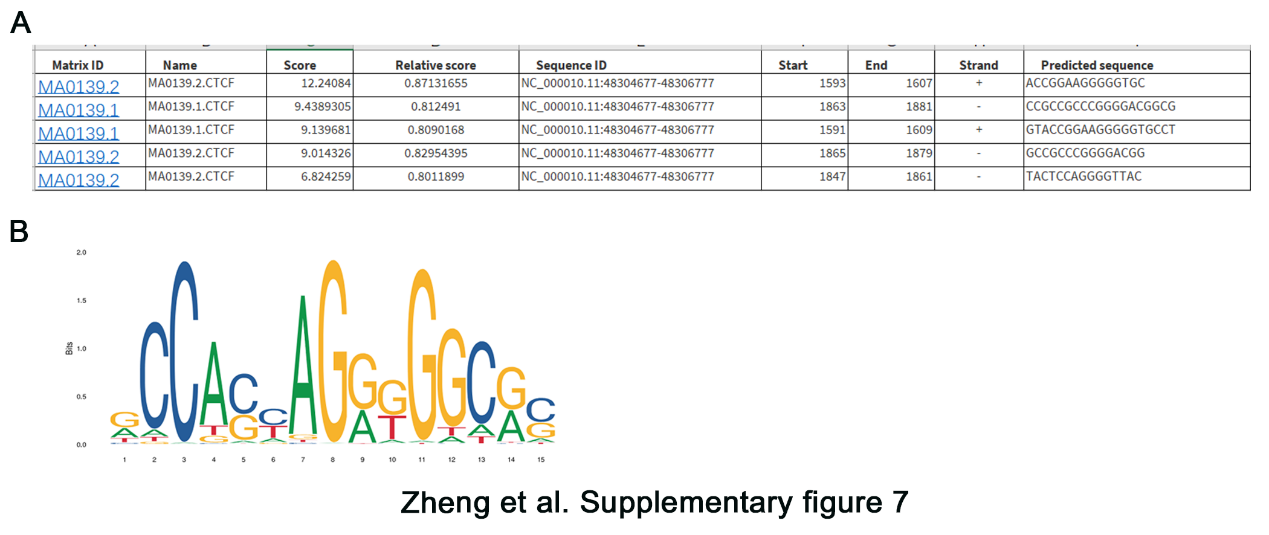


**Figure S7** Predicted aligned sequences where CTCF binds to the promoter region of JNK1 based on JASPAR database analysis (https://jaspar.elixir.no/).


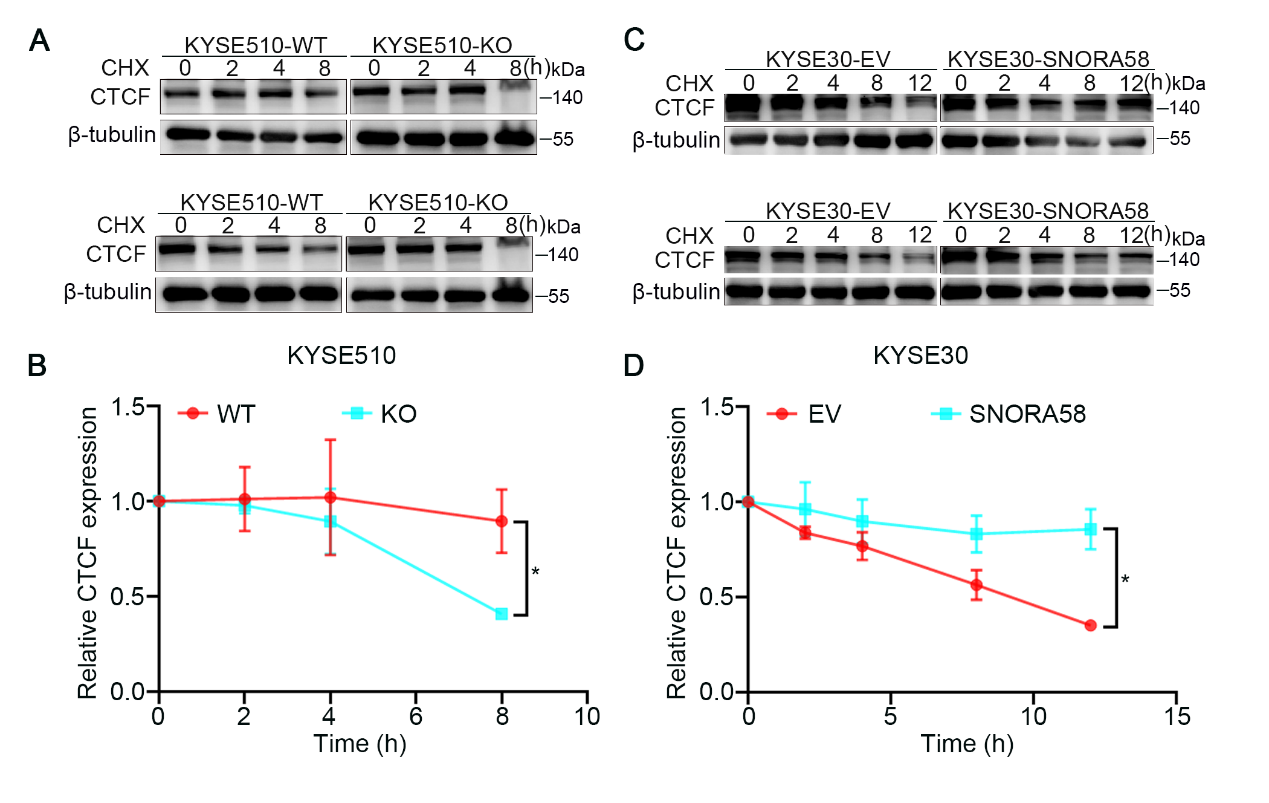


**Figure S8** The protein degradation assays and degradation curves of CTCF in SNORA58 knockout KYSE510 cells, KYSE30 cells with overexpression, and their respective control cells. CHX, Cycloheximide; WT, wild-type; KO, knockout; EV, empty vector; **P* < 0.05.


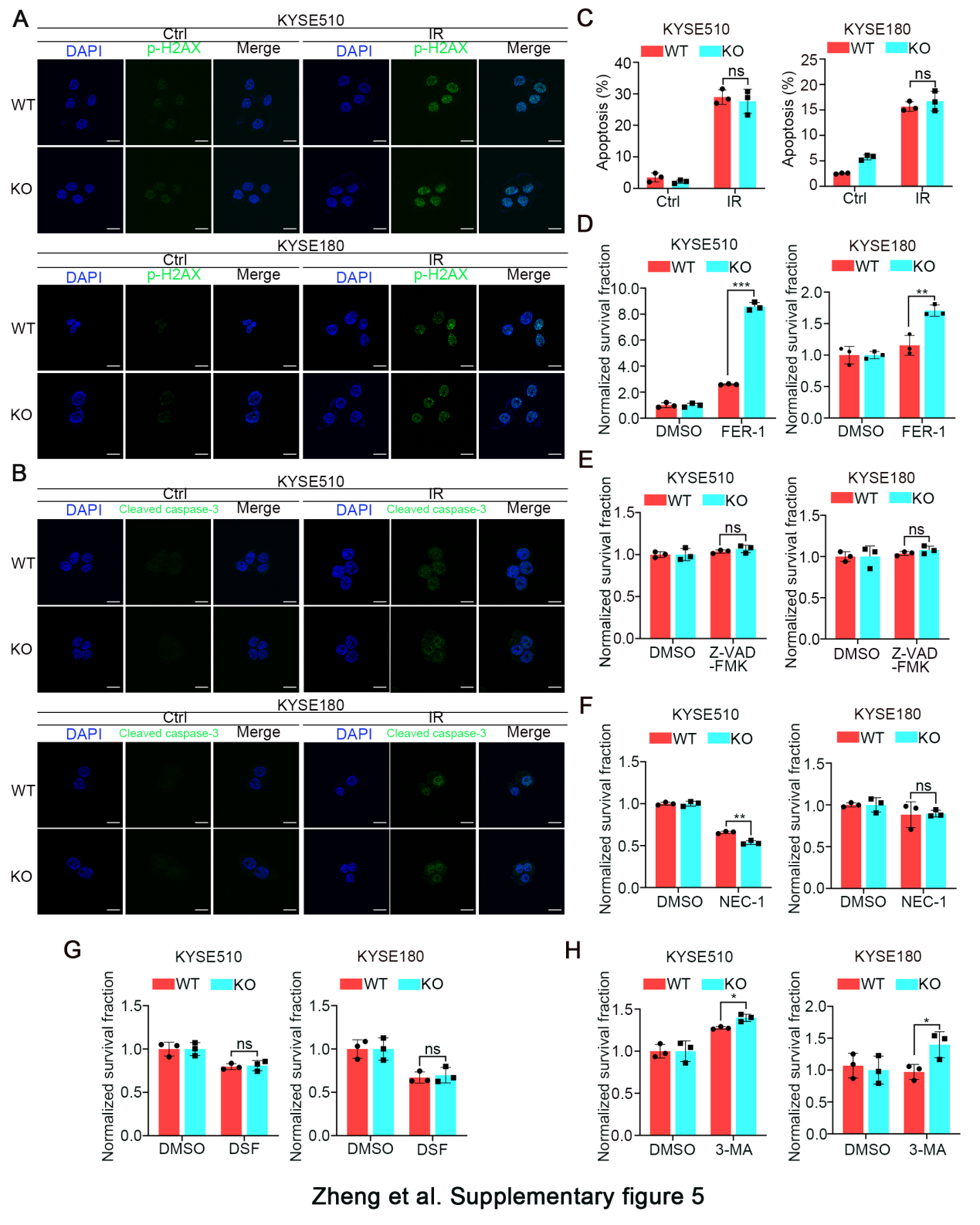


**Figure S9** SNORA58 enhances radioresistance prominently by modulating ferroptosis in ESCC cells. (**A**-**B**) The representative images of expression of the phosphorylated H2AX (**A**) and cleaved caspase-3 (**B**) detected by immunofluorescence in SNORA58 knockout cells and wild-type cells before and after exposure to10 Gy of IR. Scale bar, 20 µm**.** (**C**) The apoptosis of SNORA58 knockout cells and wild-type cells was measured by flow cytometry before and after exposure to 10 Gy of IR. (**D**-**H)** Clonogenic survival assay in SNORA58 knockout cells and wild-type cells that were pretreated with ferrostatin-1(**D**), Z-VAD-FMK (**E**), necrostatin-1 (**F**), DSF(**G**), 3-MA (**H**) or DMSO for 24 h followed by exposure to 6 Gy of IR. The survival data were normalized to those of unirradiated control cells. The data are expressed as the mean ± SD of three biological replicates and analyzed by unpaired *t-test*. **P* < 0.05, ***P* < 0.01, ****P* < 0.001. Ctrl, control; IR, ionizing radiation; WT, wild-type; KO, knockout; FER-1, ferrostatin-1; NEC-1, necrostatin-1; DSF, Disulfiram; 3-MA, 3-Methyladenine; DMSO, Dimethyl sulfoxide; ns, nonsignificant.


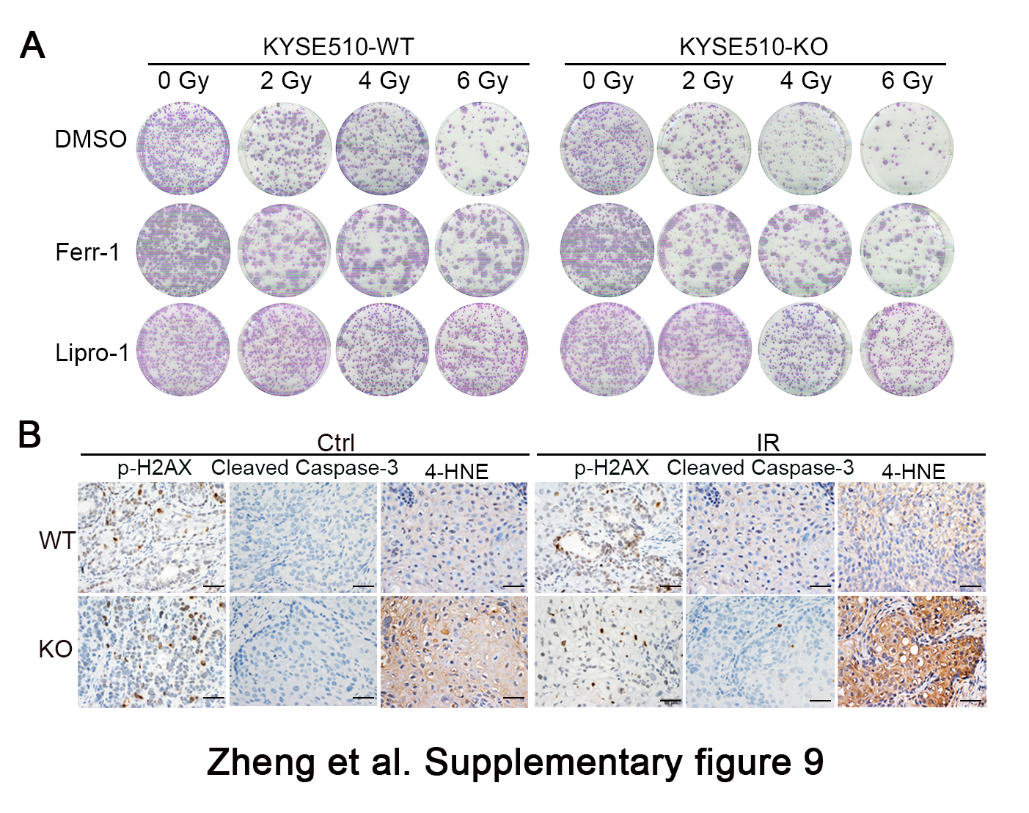


**Figure S10** SNORA58 inhibits cell ferroptosis to facilitate radioresistance. (**A**)

Representative images of clonogenic survival assay in the indicated cells pretreated with 6 μM ferrostatin-1, 8 μM liproxstatin-1 or DMSO for 24 h followed by exposure to 10 Gy of IR. (**B**) Representative images of H&E and IHC staining of phospho-H2AX, cleaved caspase-3 and 4-HNE in SNORA58 knockout cells and wild-type cells xenograft tumors with and without IR. Scale bars, 50 µm. Ctrl, control; IR, ionizing radiation; WT, wild-type; KO, knockout.


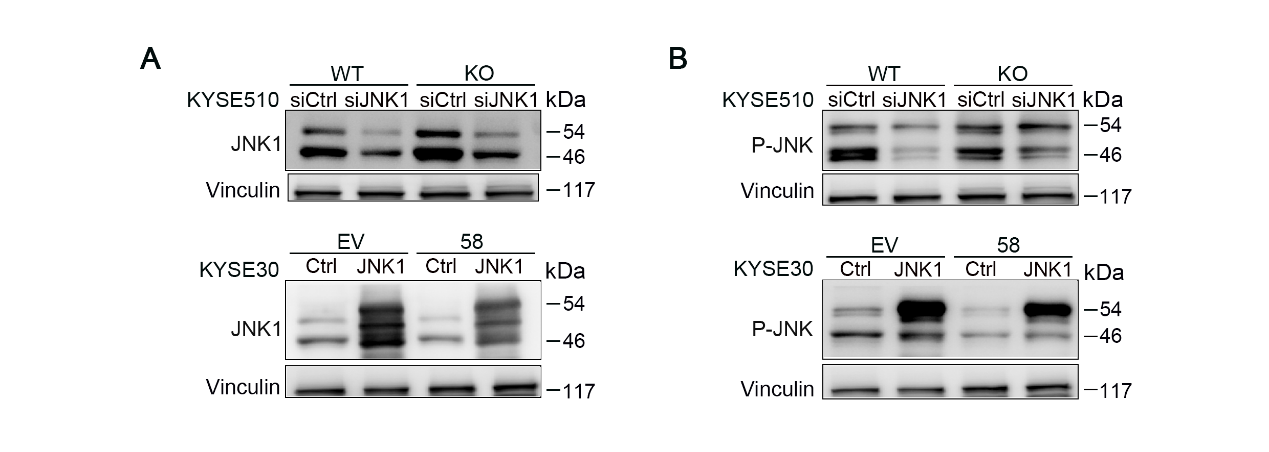


**Figure S11** Western blot analysis of the expression levels of JNK1 and phosphorylated JNK in the indicated cells. WT, wild-type; KO, knockout; EV, empty vector; 58, SNORA58.


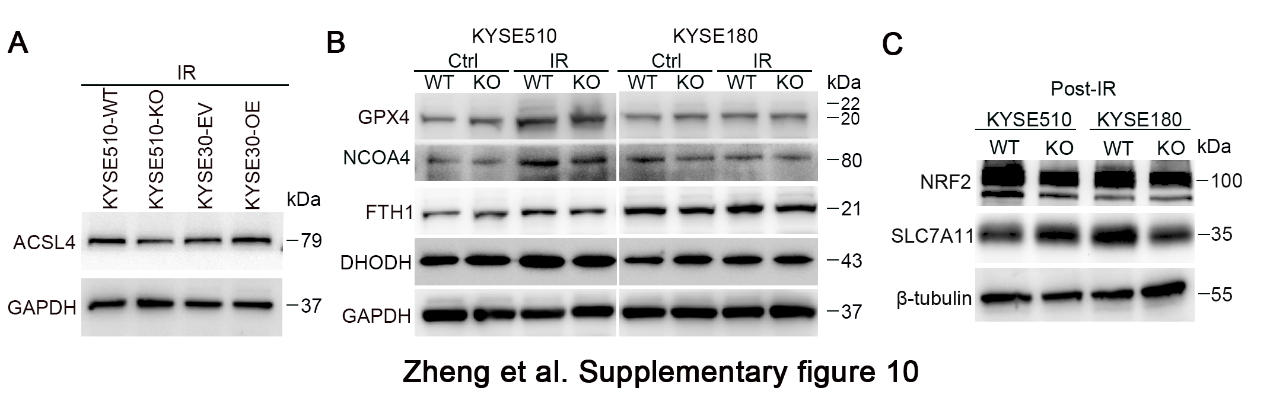


**Figure S12** SNORA58 participates in IR-induced ferroptosis not through the canonical pathways of ferroptosis reported by previous studies. (A) Western blot assay was used to detect the expression of ACSL4 in SNORA58 knockout KYSE510 cells, SNROA58 expressing KYSE30 cells and the corresponding control cells after exposure to 10 Gy of IR. (B) Western blot assay was used to detect the expression of GPX4, NCOA4, FTH1 and DHODH in SNORA58 knockout KYSE510, KYSE180 cells and the corresponding control cells after exposure to 10 Gy of IR. (C) Western blot assay was used to detect the expression of NRF2 and SLC7A11 in SNORA58 knockout KYSE510, KYSE180 cells and the corresponding control cells after exposure to 10 Gy of IR. Ctrl, control; IR, ionizing radiation; WT, wild-type; KO, knockout; OE, overexpression.


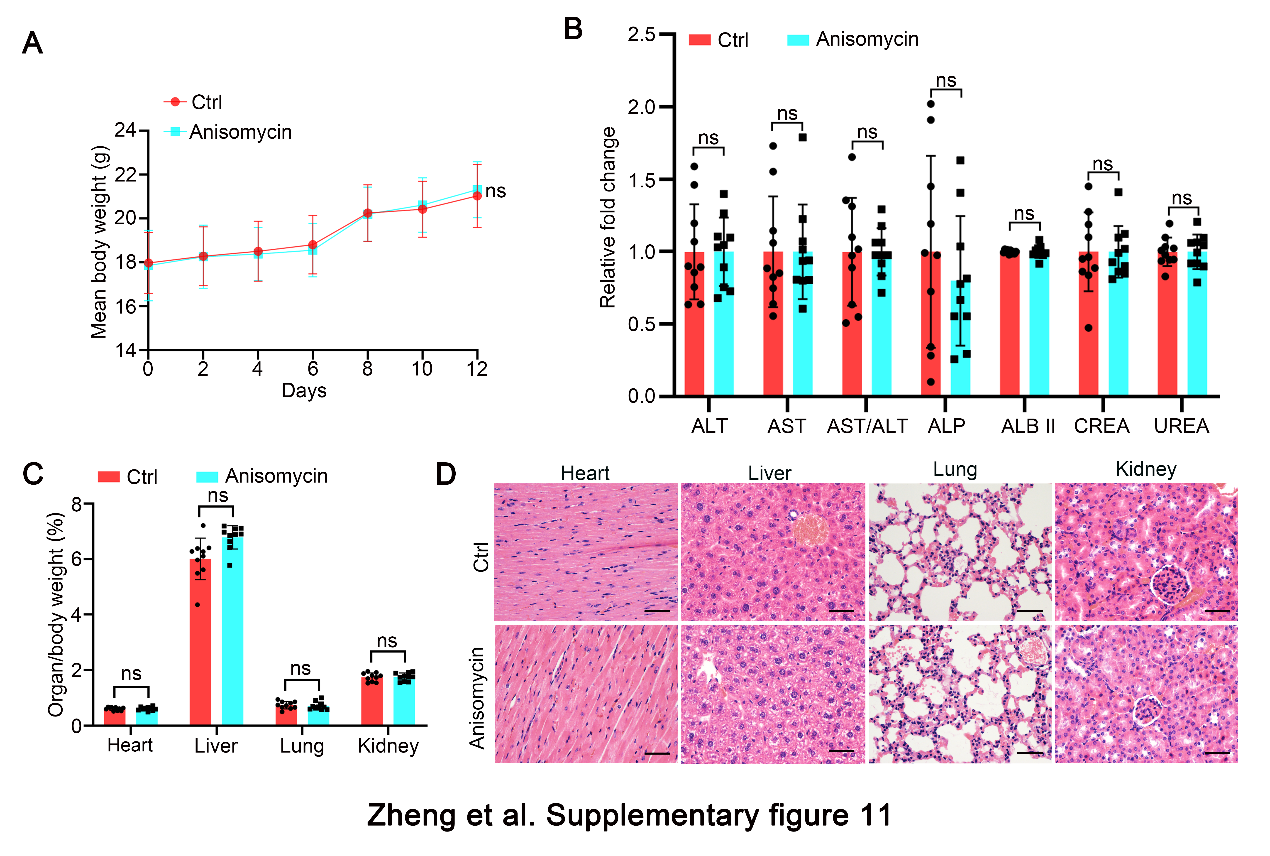


**Figure S13** Anisomycin in vivo safety evaluation. (A) Body weight changes in mice during the drug toxicology study. Mice were divided into a normal saline control group and an anisomycin-treated group (n=10 mice for each group). (B) Detection of key biochemical and metabolic markers in liver and kidney function during mouse drug toxicology experiment in the indicated groups were exhibited. (C) Comparison of terminal heart, liver, lung, and kidney weights in mice from the indicated groups in the drug toxicology experiment. (D) Representative H&E-stained images of terminal heart, liver, lung, and kidney tissues from the indicated groups in the drug toxicology experiment, Scale bars, 50 µm. The data are presented as the mean ± SD, n = 10 independent repeats and analyzed by two-way ANOVA (A) and unpaired *t*-test (B-C); ns, nonsignificant.


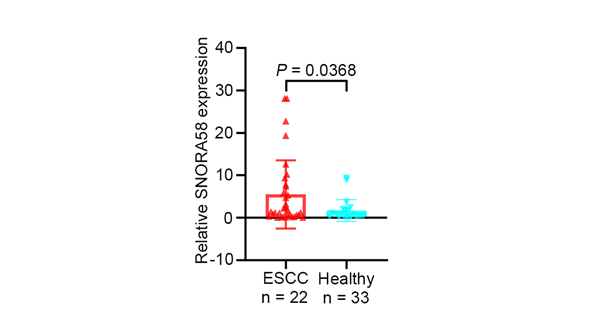


**Figure S14** The expression of SNORA58 in plasma of ESCC patients and healthy donors was quantified by qRT-PCR. The data is analyzed by unpaired t-test.


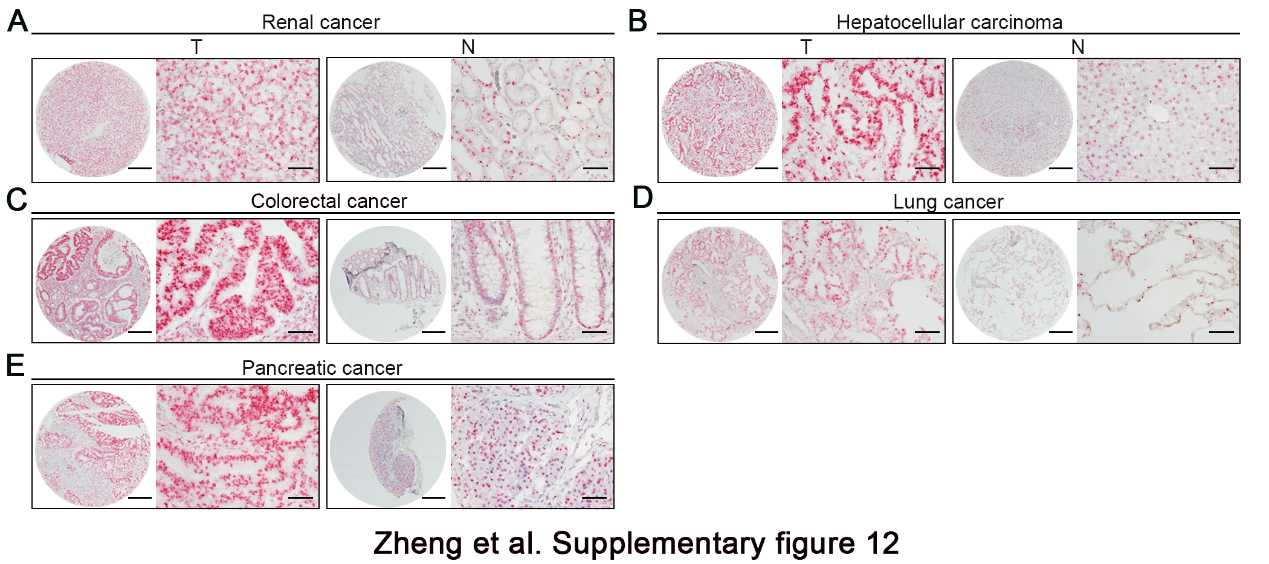


**Figure** **S15** Representative images of SNORA58 expression in indicated cancers and corresponding nontumor samples. Scale bars, left panel: 200 µm; right panel: 50 µm. T, tumor; N, nontumor.

**Supplementary Table 1. Sequences of primers used in qRT-PCR assay**

| **Gene** | **Forward primer (5'-3')** | **Reverse primer (5'-3')** |  |
| --- | --- | --- | --- |
| SNORA58 | TACTCGTAGACCTTGCCTGACT | TGATGAAGCTGGTTAGAGCTGG |  |
| JNK1 | GACGCCTTATGTAGTGACTCGC | TCCTGGAAAGAGGATTTTGTGGC |  |
| JNK2 | TACGTGGTGACACGGTACTACC | CACAACCTTTCACCAGCTCTCC |  |
| JNK3 | GTGTGGAAGTGGGAGACTCAAC | GTCAAGGACAGCATCATACGCG |  |
| CTCF | GACCACACAAGTGCCATCTCTG | ATGTCGCAGTCTGGGCACTTGT | |
| PTGS2 | CTGGCGCTCAGCCATACAG | CGCACTTATACTGGTCAAATCCC | |
| GAPDH | GTCTCCTCTGACTTCAACAGCG | ACCACCCTGTTGCTGTAGCCAA |  |
| 5s rRNA | CATACCACCCTGAACGCG | CTACAGCACCCGGTATTCCC |  |

**Plasmid sequences of JNK1 promoter wildtype and mutant**

1. JNK1-promoter-wildtype

taatgcaaggaatctgggaagaggtggatttgcaaggaatctcggaggaggtggattttggaaaaactggaggcagcttttcccgggcagctgaagagaacacttatctctctgtaggtttctgggacccggacaggtgtcctcagaatggaacatattgtcggtcaaggtaaccagctcttcatcactcaggtgtgtgtaccggaagggggtgcctgttcgctgcgcaggttaggcctaagcgcacctgcccttcaaccctgtgccgacgaggcccgaaggcttaagctctgacctggctaccgccgccaaccagatgcggtcccctggggccacgcgccgccacgtgccgccacagggcccgcccctcccgctccaggccacgcccctcacccacagccctcggaggctctct

1. JNK1-promoter-mutant (The mutant sites have been highlighted)

taatgcaaggaatctgggaagaggtggatttgcaaggaatctcggaggaggtggattttggaaaaactggaggcagcttttcccgggcagctgaagagaacacttatctctctgtaggtttctgggacccggacaggtgtcctcagaatggaacatattgtcggtcaaggtaaccagctcttcatcactcaggtgtgtgtTGGCCTTCCCCCACGctgttcgctgcgcaggttaggcctaagcgcacctgcccttcaaccctgtgccgacgaggcccgaaggcttaagctctgacctggctaccgccgccaaccagatgcggtcccctggggccacgcgccgccacgtgccgccacagggcccgcccctcccgctccaggccacgcccctcacccacagccctcggaggctctct

**Supplementary Table 2.** The information of Antibodies used in this study

| **Primary antibodies** | **Application** | **Vender** | **Catalog#** |
| --- | --- | --- | --- |
| Ki67 | IHC, 1:100 | ZSBG | ZA-0502 |
| 4-Hydroxynonenal | IHC, 1:200 | Abcam | ab46545 |
| Cleaved caspase-3 (Asp175) | IHC, 1:200  IF, 1:50 | CST | 9661 |
| JNK1 | IHC, 1:200 | Invitrogen | 44-690G |
| Phospho-SAPK/JNK (Thr183/Tyr185) | IHC, 1:100  WB, 1:1000 | CST | 9251 |
| Phospho-Histone H2A.X (Ser139) | IHC, 1:200  IF, 1:1000 | CST | 2577 |
| JNK | WB, 1:1000 | CST | 9252 |
| JNK1 | WB, 1:1000 | CST | 3708 |
| Phospho-p38 (Thr180/Tyr182) | WB, 1:1000 | CST | 4511 |
| p38 | WB, 1:1000 | CST | 9212 |
| p44/42 (Erk1/2) | WB, 1:1000 | CST | 4695 |
| Phospho-p44/42 (Erk1/2) (Thr202/Tyr204) | WB, 1:1000 | CST | 4370 |
| CTCF | WB,1:1000  CHIP,5 μg/test  RIP, 5 μg/test  IP,4 μg/test | Abcam | 128873 |
| Ubiquitin (P4D1) | WB,1:200 | Santa Cruz | sc-8017 |
| Ferritin light chain | WB, 1:1000 | Proteintech | 10727-1-AP |
| ACSL4 | WB, 1:1500 | Abcam | 155282 |
| GPX4 | WB, 1:1000 | CST | 52455 |
| NCOA4 | WB, 1:1000 | CST | 66849 |
| FTH1 | WB, 1:1000 | CST | 4393 |
| DHODH | WB, 1:1000 | Proteintech | 14877-1-AP |
| Vinculin | WB, 1:1000 | Proteintech | 26520-1-AP |
| GAPDH | WB, 1:1000 | CST | 2118 |
| **Secondary antibodies** | **Application** | **Vender** | **Catalog#** |
| Alexa Fluor 488-labeled Goat Anti-Rabbit IgG(H+L) | IF, 1:1000 | Beyotime | A0423 |
| Anti-rabbit IgG, HRP-linked Antibody | WB, 1:1000 | CST | 7074 |
| Anti-mouse IgG, HRP-linked Antibody | WB, 1:1000 | CST | 7076 |
